# Neoadjuvant treatment of pancreatic adenocarcinoma: a systematic review and meta-analysis of 5520 patients

**DOI:** 10.1186/s12957-017-1240-2

**Published:** 2017-10-10

**Authors:** Mashaal Dhir, Gautam K. Malhotra, Davendra P.S. Sohal, Nicholas A. Hein, Lynette M. Smith, Eileen M. O’Reilly, Nathan Bahary, Chandrakanth Are

**Affiliations:** 1 Department of Surgery, SUNY Upstate Medical University, Syracuse, NY 13210 USA; 2 Department of Surgery, University of Nebraska Medical Center, Omaha, NE 98198 USA; 3 Division of Hematology and Oncology, Taussig Cancer Institute, Cleveland Clinic, Cleveland, OH 44195 USA; 4 Department of Biostatistics, College of Public Health, University of Nebraska Medical Center, Omaha, NE 68198 USA; 5 David M. Rubenstein Center for Pancreatic Cancer, Memorial Sloan Kettering Cancer Center, New York, NY 10065 USA; 6 Department of Medicine, Division of Hematology and Oncology, University of Pittsburgh Medical Center, Pittsburgh, PA 15232 USA; 7 Department of Surgery, Division of Surgical Oncology, University of Nebraska Medical Center, Omaha, NE 98198 USA; 8 Department of Surgery/Genetics, Cell Biology and Anatomy, University of Nebraska Medical Center, Omaha, NE 68198 USA

**Keywords:** Pancreatic cancer, Pancreatic adenocarcinoma, Neoadjuvant therapy, Outcomes, Survival

## Abstract

**Background:**

Recent years have seen standardization of the anatomic definitions of pancreatic adenocarcinoma, and increasing utilization of neoadjuvant therapy (NAT). The aim of the current review was to summarize the evidence for NAT in pancreatic adenocarcinoma since 2009, when consensus criteria for resectable (R), borderline resectable (BR), and locally advanced (LA) disease were endorsed.

**Methods:**

PubMed search was undertaken along with extensive backward search of the references of published articles to identify studies utilizing NAT for pancreatic adenocarcinoma. Abstracts from ASCO-GI 2014 and 2015 were also searched.

**Results:**

A total of 96 studies including 5520 patients were included in the final quantitative synthesis. Pooled estimates revealed 36% grade ≥ 3 toxicities, 5% biliary complications, 21% hospitalization rate and low mortality (0%, range 0–16%) during NAT. The majority of patients (59%) had stable disease. On an intention-to-treat basis, R0-resection rates varied from 63% among R patients to 23% among LA patients. R0 rates were > 80% among all patients who were resected after NAT. Among R and BR patients who underwent resection after NAT, median OS was 30 and 27.4 months, respectively.

**Conclusions:**

The current study summarizes the recent literature for NAT in pancreatic adenocarcinoma and demonstrates improving outcomes after NAT compared to those historically associated with a surgery-first approach for pancreatic adenocarcinoma.

**Electronic supplementary material:**

The online version of this article (10.1186/s12957-017-1240-2) contains supplementary material, which is available to authorized users.

## Background

Pancreatic cancer is one of the leading causes of cancer-related mortality with an estimated 53,670 new cases and an estimated 43,090 deaths in 2017 [1]. The overall survival for all patients with pancreatic cancer remains dismal—approximately 7% at 5 years [2–4]. Most of the patients present with metastatic disease. Even for those without metastatic disease, surgical treatment can be complex given the local extent of the tumor, with only 10–20% being candidates for upfront surgery, and these operable patients may experience 5 year survival of 10–30% [3]. Recent years have seen several advances in the management of pancreatic adenocarcinoma. In 2009, an expert consensus statement defined the criteria (based on vascular involvement assessed by preoperative imaging) based on which non-metastatic pancreatic adenocarcinoma is broadly categorized into three categories: resectable, borderline resectable and locally advanced disease [5–7]. Although subtle differences remain between the different consensus criteria (NCCN, Intergroup, AHPBA/SSO, etc.) based on the extent of vascular involvement, these definitions have made the anatomic classification of pancreatic adenocarcinoma more uniform and have made cross study comparisons more reliable.

Since 1997, gemcitabine has been the reference drug for advanced pancreatic cancer after a randomized trial reported a marginal improvement in overall survival and improved quality of life over 5-FU alone (5.6 vs 4.4 months, *p* = 0.0025) [8]. Systemic therapy for advanced pancreatic adenocarcinoma (including metastatic disease) has improved with the availability of more effective chemotherapeutic regimens such as FOLFIRINOX (median OS 11.1 months, RR 31.6%) and gemcitabine with nab-paclitaxel (median OS 8.5 months, RR 23%). [9, 10] Advances in the chemotherapeutic treatment of metastatic pancreatic adenocarcinoma have been extrapolated to other potentially resectable categories of pancreatic adenocarcinoma [9–15]. More aggressive multimodality treatments have been employed for the management of resectable and borderline resectable tumors in addition to locally advanced tumors [11–13]. Imaging modalities (pancreas protocol CT scan) and radiation delivery techniques such as IMRT and SBRT have evolved which have further advanced the care of these patients.

Neoadjuvant treatment (NAT) strategies are increasingly being employed for borderline resectable and resectable tumors [11–13]. These strategies lead to early initiation of systemic therapy in these patients in contrast to a surgery first approach where up to half of the patients may not receive adjuvant therapy due to postoperative complications or decline in functional status [16–18]. Theoretically, the neoadjuvant approach downstages nodal disease, increases the rate of margin negative resection, and also helps to identify patients at risk of early disease progression [19, 20].

Although previous authors have elegantly reviewed the literature on the subject, recent years have seen several advancements as mentioned above [21–23]. The aim of the current study was to perform a systematic review of the literature since 2009 (the year in which the expert consensus criteria were endorsed by several surgical societies) and summarize the current landscape for the role and outcomes of neoadjuvant therapy in the treatment of all non-metastatic anatomic subcategories of pancreatic cancer, i.e., resectable, borderline resectable and locally advanced unresectable disease.

## Methods

### Search strategy

Articles in PubMed database from January, 2009 to May 2015 were searched using the key words “Neoadjuvant” [All Fields] AND “Pancreatic cancer” [All Fields]. Such a search strategy was chosen to capture any English language article reporting on the neoadjuvant treatment of pancreatic cancer. PubMed database was used as it remains one of the most widely used medical literature resources, and only indexes peer-reviewed biomedical literature [24].

### Inclusion and exclusion criteria

**Fig. 1:**
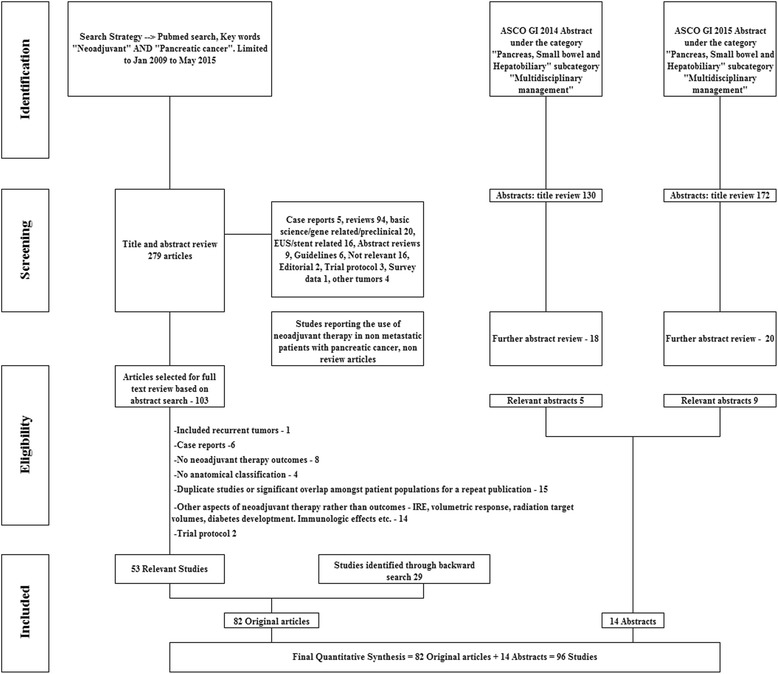
PRISMA Study flow diagram depicting search strategy, screening, selection, and exclusion criteria

Title and abstract review was performed for 279 articles to identify relevant articles. Studies reporting the use of neoadjuvant therapies in non-metastatic patients with pancreatic adenocarcinoma were selected for full text review. Case reports, letters, and review articles were excluded. The search strategy is depicted in detail in Fig. 1. Prospective or retrospective studies of patients with pancreatic adenocarcinoma treated with neoadjuvant therapies (including chemotherapy, chemoradiotherapy or radiotherapy) were evaluated. All phase 1–2 clinical trials, cohort studies and case series were included. Only studies of patients with pancreatic adenocarcinoma were included. Studies reporting combined outcomes of periampullary tumors were excluded. All identified case reports and studies with identical patient cohorts over a similar time period were excluded. A backward search was also performed using bibliographies of relevant articles and review articles to ensure a comprehensive search.

Additionally, abstracts from American Society of Clinical Oncology, Gastrointestinal Cancer Symposium (ASCO GI) under the categories “Cancers of the Pancreas, Small Bowel, and Hepatobiliary Tract” AND “Multidisciplinary Treatment” were reviewed. Title review was performed for 302 abstracts of which 38 were further selected for abstract and review of online posters. Fifteen relevant abstracts were included in the qualitative and quantitative synthesis. Search for conference abstracts was limited as there may be differences in the data presented in the initial abstract and final publication [25].

Figure 1 depicts the study flow diagram. Preferred Reporting Items for Systematic Reviews and Meta-analysis (PRISMA) guidelines were followed with regards to reporting of results [26]. A checklist has been included in the supplementary files.

### Data extraction and definitions

Data were extracted from the selected articles by the first and second author (M.D. and G.M.). Inconsistencies were resolved by discussion and consulting the corresponding author when needed. Data extracted included first author, article type, journal, study methodology, type of neoadjuvant therapy, regimen details, type of tumors (resectable, borderline resectable, locally advanced or combinations thereof), number of patients, radiologic response (complete response, partial response, stable disease, progression), overall and grade 3 toxicity of neoadjuvant therapy, grade 3 toxicities of specific types (i.e., anemia, leukopenia, lymphopenia, neutropenia, febrile neutropenia, thrombocytopenia, fatigue, nausea/vomiting, diarrhea, anorexia, weight loss, biliary obstruction/cholangitis, gastritis/duodenitis/GI bleed), readmission rates, mortality associated with neoadjuvant therapy, pathologic response, overall survival, and progression-free survival.

For the calculation of toxicities, radiologic response, and resection rates only studies reporting the outcomes of all patients who underwent neoadjuvant therapy followed by restaging were included. Studies reporting the outcomes of only patients resected after neoadjuvant therapy were included in the calculation of postoperative morbidity and mortality. For studies reporting on combination of various anatomic categories, e.g., borderline resectable and locally advanced, data were recorded separately wherever feasible. When different anatomic subcategories could not be used separately for data extrapolation due to reporting of combined outcomes such studies were listed under BR + LA (borderline resectable and locally advanced patients) and R + BR + LA (resectable, borderline resectable, and locally advanced). Only BR + LA category results were reported given the limited number of patients in the other combined categories. Similarly, for double-armed studies or retrospective studies reporting on two different treatment types, data were recorded separately wherever feasible. The data extraction process was performed twice to ensure the accuracy of the data. Data were also recorded on vascular resections especially for BR and LA categories, dose of radiation therapy, age and gender of the study population, number of cycles of chemotherapy, adjuvant therapy, and follow up to assess the quality of the studies. However, given lack of consensus to assess the quality of non-randomized clinical trials, all studies were included based on completeness of data and inclusion/exclusion criteria.

### Statistical methods

Summary statistics are reported as total and percentages for categorical variables vs mean or median with corresponding standard deviation or ranges, respectively, for continuous variables. Meta-analysis was performed using STATA 14.2 statistical software (StataCorp, 4905 Lakeway Drive, College Station, Texas). Meta-analysis of proportions strategy using the command “metaprop” was utilized. Pooled estimates of proportions with corresponding 95% confidence intervals were reported and calculated using Freeman-turkey double arcsine transformation [27]. Random effects model was used due to our suspicion of heterogeneity among different studies. Heterogeneity was explored using the chi-squared test with a significance level of *p* = 0.05. *I* ^2^ was calculated to further quantify heterogeneity. Publication bias was explored using funnel plots and symmetry of the funnel plot was analyzed using visual inspection and Begg’s tests to rule out any bias from the studies with small patient samples [28, 29]. Additionally, subset analyses were used wherever feasible. The weighted aggregated median survival times were generated using SAS software, Version 9.4 (SAS Institute Inc., Cary, NC, USA). Since most studies only supplied median survival times and no additional information, i.e., patients at risk, confidence intervals, hazard ratios, etc. were consistently available, we used the method described by Gillen et al. to weight and aggregate the median survival times [23]. Briefly, *w*_*i*_ denotes the weight of an individual study which is calculated as *n*/*N* where *n* is the number of study participants, and N is the total number of evaluable patients within a stratum. If *m*_*i*_ (*i* = 1, …, *k*) represents the median survival time of an individual study within a stratum, then a weighted estimate of the median survival of the stratum (*m*_*s*_) can be calculated as follows.

$$ {m}_s={\left(\sum_{i=1}^k\frac{w_i}{m_i}\right)}^{-1} $$ where ∑*w*_*i*_ = 1.

Confidence intervals cannot be computed using the method described by Gillen et al. [23]. Instead, Gillen et al. recommend reporting the minimum and maximum median survival times. [23]

## Results

### Patient and study characteristics

**Fig. 2:**
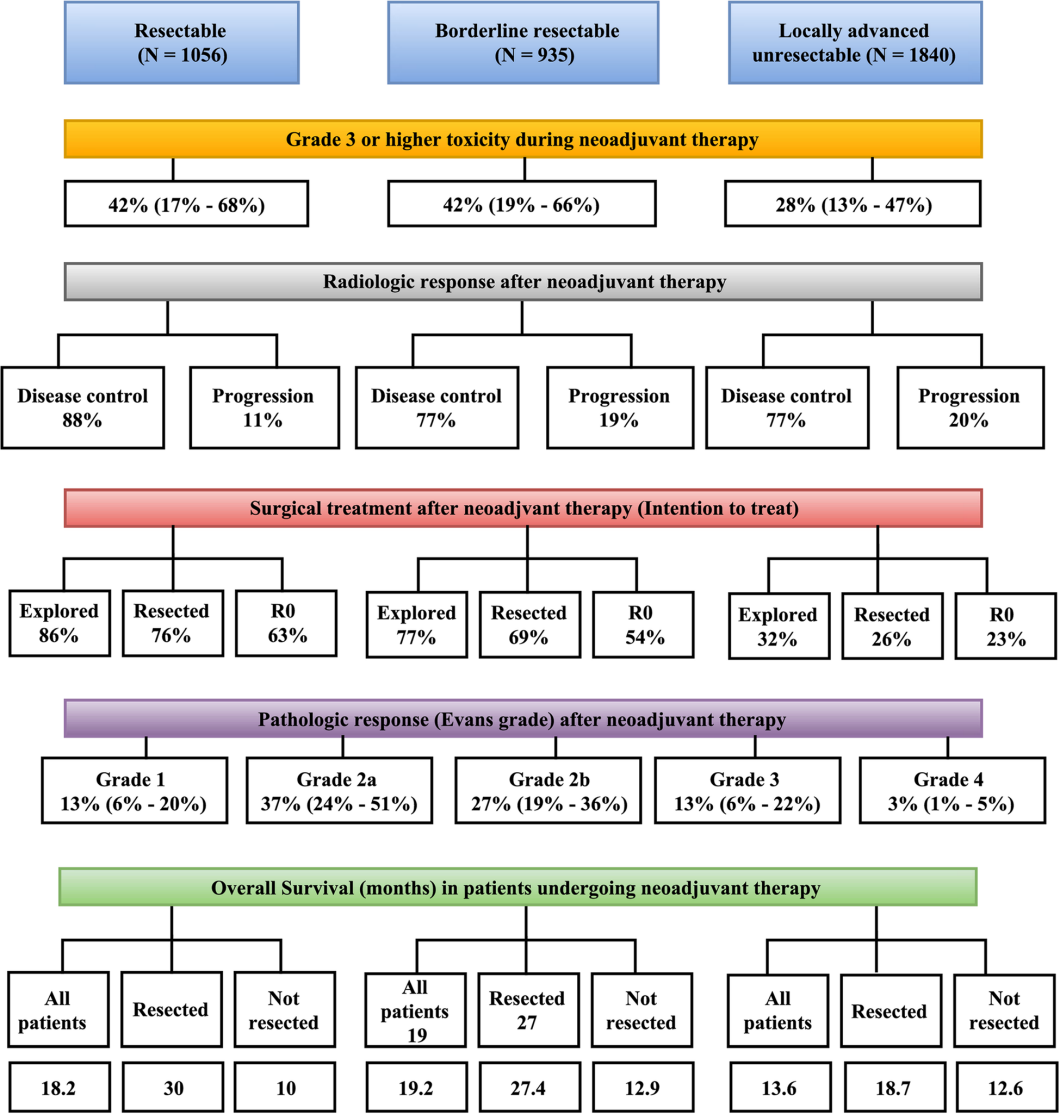
Summary of outcomes after neoadjuvant therapy among resectable, borderline resectable, and locally advanced unresectable patients with pancreatic adenocarcinoma

A total of 96 studies [30–125] including 82 original articles and 14 abstracts including 5520 patients, were selected for qualitative and quantitative synthesis. These include 1056 patients with resectable tumors, 935 with borderline resectable tumors, and 1840 with locally advanced unresectable tumors, with an additional 1689 patients in overlapping categories. Most of the studies were retrospective series but 22 phase II studies were also included in the current analysis. Sixty-five percent of the included studies were from 2013 to 2015. Majority (78.1%) of the studies mentioned resectability criteria. Majority of the included studies were single institutional (86.5%) and majority were from institutions located in North America and Europe (78.2%). Chemotherapy alone was used in 20 (20.8%) studies, chemoradiotherapy in 33(34.4%) studies, chemotherapy with chemoradiation was used in 41 (42.7%), and radiation alone was used in 2 (2.1%) studies. Type of chemotherapy agent utilized included FOLFIRINOX in 810 patients, GTX in 410 patients, single drug (gemcitabine/5FU/capecitabine) in 1521 patients, two-drug regimen (gem + cisplatin/nab-paclitaxel/oxaliplatin/bevacizumab/docetaxel/S1) in 1113 patients or three drug combinations other than FOLFIRINOX or GTX in 60 patients. Studies with 1198 patients described use of multiple regimes whereas 222 patients had RT alone and in 186 patients chemotherapy agent was not described. Table 1 provides a summary of major characteristics of the included studies and Additional file 1: Table S1 provides details on all the included studies. Figure 2 provides a snapshot and Tables 1 through 5 provide details of results which are also elaborated below.

**Table 1:** Summary of studies included in the quantitative analysis

			*N*	%
Total number of studies included			96	
		Original articles	82	85.4
		Abstracts	14	14.6
Year of publication				
		2009	6	6.3
		2010	7	7.3
		2011	8	8.3
		2012	12	12.5
		2013	17	17.7
		2014	29	30.2
		2015	17	17.7
Global distribution of location of institutions of selected studies				
		North America	54	56.3
		Europe	21	21.9
		Asia	18	18.7
		Australia/Africa/South America (1 each)	3	3.1
Single vs multi-institutional				
		Single institution	83	86.5
		Multi-institutional	13	13.5
Type of study				
		Retrospective series	54	56.3
		Phase 2 studies	22	22.9
		Prospective series	11	11.5
		Phase 1 studies	5	5.2
		Phase 1/2 studies	3	3.1
		Phase 3 studies	1	1
Criteria for resectability				
		NCCN/AHPBA/SSO/SSAT consensus	38	39.6
		MD Anderson	8	8.3
		Other	29	30.2
		Not mentioned	21	21.9
Number of studies of each local/anatomical stage				
		Resectable	18	18.8
		Borderline resectable (BR)	15	15.6
		Locally advanced unresectable (LA)	29	30.2
		R + BR	5	5.2
		BR + LA	24	25
		R + BR + LA	5	5.2
Number of patients in each anatomical stage				
		Resectable	1056	19.1
		Borderline resectable (BR)	935	16.9
		Locally advanced unresectable (LA)	1840	33.3
		R + BR	202	3.7
		BR + LA	1014	18.4
		R + BR + LA	473	8.6
Criteria for of radiology response assessment				
		RECIST	48	50
		WHO	5	5.2
		Other	2	2.1
		Not mentioned	41	42.7
Criteria for toxicity assessment				
		NCI CTCAE	43	44.8
		WHO	5	5.2
		RTOG	2	2.1
		Not mentioned/not applicable	46	47.9
Type of neoadjuvant therapy				
		Chemoradiotherapy	33	34.4
		Chemotherapy alone	20	20.8
		Chemotherapy with chemoradiation	41	42.7
		Radiation alone	2	2.1
Chemotherapy drugs				
		Monotherapy (Gem/5FU/Cape/UFT/Cis)	26	27.1
		FOLFIRINOX	20	20.8
		Gemcitabine + oxaliplatin*	8	8.3
		Gemcitabine or 5FU + cisplatin	7	7.3
		Gemcitabine + Docetaxel	3	3.1
		GTX (gemcitabine, taxane, capecitabine)	6	6.3
		Gem + biologic (bevacizumab, cetuximab)	3	3.1
		Gem + S1	5	5.2
		Gem + Nab-paclitaxel	2	2.1
		5FU + cisplatin + interferon	1	1
		Multiple	11	11.5
		Not mentioned	2	2.1
		None	2	2.1
Radiotherapy				
		Yes	76	79.2
		No	20	20.8

*Includes one study with gemcitabine + oxaliplatin + cetuximab

*R* resectable, *BR* borderline resectable, *LA* locally advanced, *Gem* Gemcitabine, *Cape* capecitabine, *5FU* 5 Fluorouracil, *UFT* uracil/tegafur

### Toxicity

Only grade ≥ 3 toxicities were analyzed. Data regarding any form of grade ≥ 3 toxicity were available in 55 of the 96 studies. Details regarding toxicity are summarized in Table 2. Briefly, overall grade ≥ 3 toxicity was seen in 36% of the patients (95% CI 27–45%). Hematologic grade ≥ 3 toxicity was more common than non-hematologic grade ≥ 3 toxicity (25 vs 16%). One-fifth of all patients receiving therapy were hospitalized during the neoadjuvant therapy. Over 90% of the patients were able to complete majority of the neoadjuvant treatment (not considering dose reductions). Most studies reported a mortality rate of 0% (95% CI 0–1%). Funnel plots for overall grade ≥ 3 toxicity are reported in Additional file 3: Figure S1. No statistically significant publication bias was noted using Begg’s test (*p* = 0.572) and Egger’s test (*p* = 0.293).

**Table 2:** Grade 3 or higher toxicities, readmission rates and mortality during the neoadjuvant treatment of patients with pancreatic adenocarcinoma

Grade 3 or higher toxicities	Number of studies reporting the studied outcome	%	95% confidence interval
Overall	39	36%	27–45%
Hematologic	16	25%	14–38%
Anemia	33	4%	3–6%
Leukopenia	26	25%	18–32%
Lymphopenia	8	13%	4–24%
Neutropenia	38	23%	17–29%
Febrile neutropenia	17	3%	1–5%
Thrombocytopenia	41	7%	5–10%
Non hematologic	17	16%	7–27%
Nausea/vomiting	40	7%	4–10%
Diarrhea	38	4%	2–6%
Anorexia	19	3%	1–6%
Fatigue	26	4%	1–7%
Biliary obstruction/cholangitis	22	5%	3–7%
GI bleed, gastritis, or duodenitis,	14	3%	1–7%
Hospitalization rate	15	21%	14–27%
% of patients completing neoadjuvant therapy	42	90%	87%–93%
Mortality	48	0%	0–1%

**Fig. 3:**
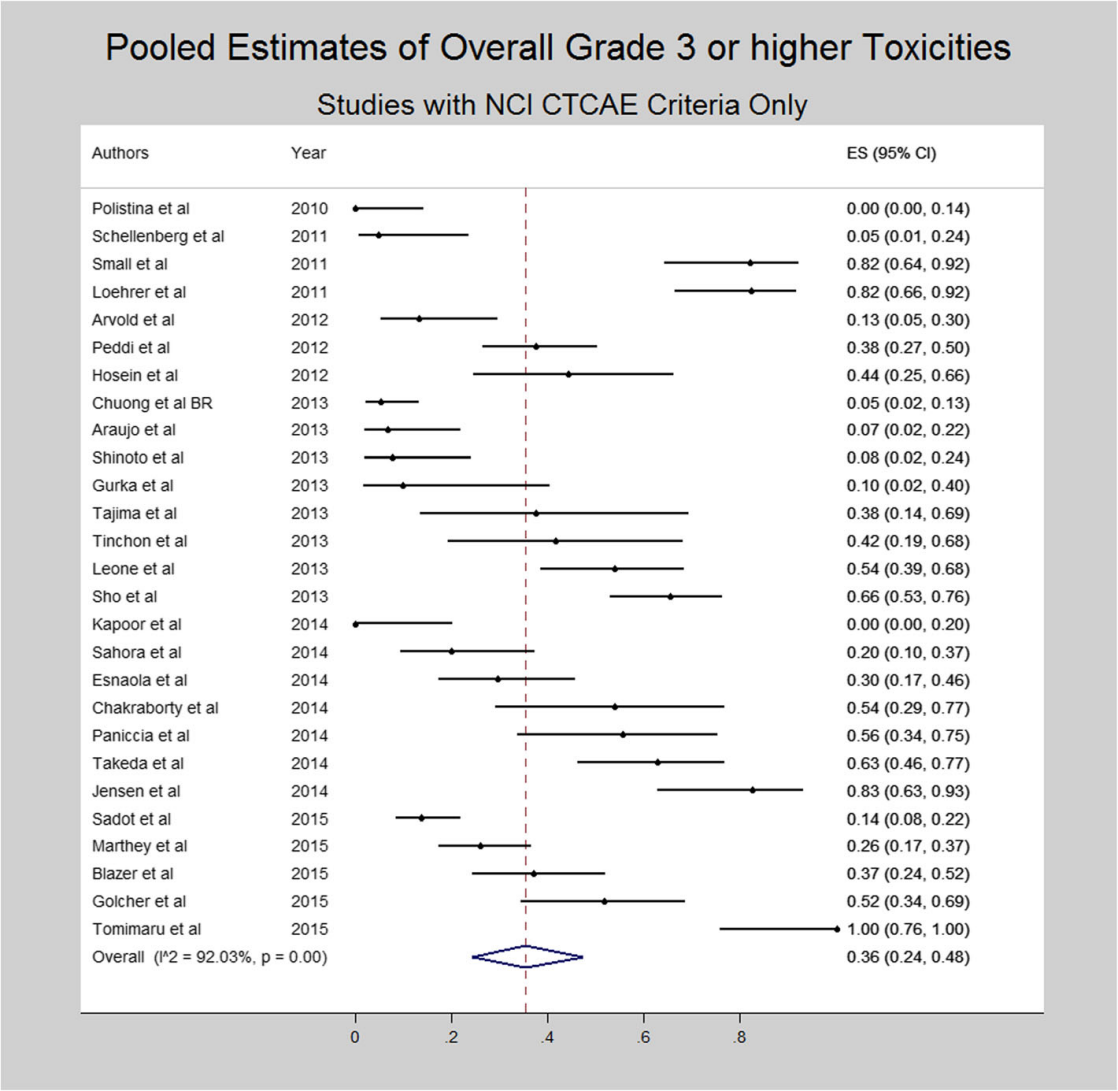
Forest plot depicting pooled estimates for overall grade ≥ 3 toxicities among studies utilizing NCI CTCAE criteria for toxicity assessment during neoadjuvant therapy

A subset analysis was performed for overall grade ≥ 3 toxicities for studies utilizing NCI CTCAE criteria. Again overall grade ≥ 3 toxicity was noted in 36% of the patients (95% CI 24–48%). Pooled estimates for studies utilizing NCI CTCAE criteria were similar to those obtained for all studies reporting overall grade ≥ 3 toxicities. Figure 3 depicts the forest plot for overall grade ≥ 3 toxicities in the studies utilizing NCI CTCAE criteria.

### Radiologic response

The results for radiologic response by broad categories (disease control and progression) are outlined in Fig. 2, whereas the complete details of radiologic response (complete response, partial response, stable disease and progressive disease) are highlighted in Table 3. Tumor response was evaluated in 61 studies prior to resection, however not all studies mentioned all response categories including complete response, partial response, stable disease and progressive disease. As previously reported, the incidence of complete response was between 0–1%. Overall partial response was seen in 20% (95% CI 16–25%). Most patients had stable disease 59% (95% CI 54–65%). Overall progressive disease during neoadjuvant therapy was noted in 16% (95% CI 12–20%) of patients.

**Table 3:** Radiologic response after completion of neoadjuvant therapy for patients with pancreatic adenocarcinoma

	Radiologic response			
	Complete response	Partial response	Stable disease	Progressive disease
Resectable	*0%*	*11%*	*77%*	*11%*
	[0–0%]	[8–16%]	[62–89%]	[4–19%]
	*I* ^2 =^ 0%	*I* ^2^ = 10.6%	*I* ^2^ = 85.12%	*I* ^2^ = 82.31%
	(*n* = 8)	(*n* = 8)	(*n* = 9)	(*n* = 11)
Borderline resectable	*1%*	*20%*	*56%*	*19%*
	[0–4%]	[8–35%]	[47–64%]	[11–28%]
	*I* ^2^ = 52.03%	*I* ^2^ = 84.28%	*I* ^2^ = 33.25%	*I* ^2^ = 58.95%
	(*n* = 8)	(*n* = 8)	(*n* = 8)	(*n* = 9)
Locally advanced	*1%*	*28%*	*48%*	*20%*
	[0–3%]	[21–35%]	[38–58%]	[13–29%]
	*I* ^2^ = 57.02%	*I* ^2^ = 82.61%	*I* ^2^ = 88.09%	*I* ^2^ = 89.43%
	(*n* = 17)	(*n* = 19)	(*n* = 17)	(*n* = 18)
BR + LA	*0%*	*20%*	*59%*	*14%*
	[0–1%]	[11–31%]	[49–69%]	[8–22%]
	*I* ^2^ = 7.57%	*I* ^2^ = 84.70%	*I* ^2^ = 76.89%	*I* ^2^ = 72.59%
	(*n* = 16)	(*n* = 16)	(*n* = 16)	(*n* = 16)
All patients	0%	*20%*	*59%*	*16%*
	[0–1%]	[16–25%]	[54–65%]	[12–20%]
	*I* ^2^ = 38.25%	*I* ^2^ = 81.52%	*I* ^2^ = 85.12%	*I* ^2^ = 82.84%
	(*n* = 52)	(*n* = 56)	(*n* = 56)	(*n* = 62)

Values in [] reflect the 95% confidence interval. *I* ^2^ is a quantitative indicator of heterogeneity among the studies, and *n* refers to the number of studies reporting the outcomes

In patients with resectable tumors, there were no complete radiologic responses. Partial response was seen in 11% of patients and majority (77%) had stable disease. Progression was noted in 11% of the patients. In patients with borderline resectable tumors, 20% had partial response, 56% had stable disease and 19% progressed during therapy. In patients with locally advanced disease, 1% had complete response, 28% had partial response, 48% stable disease, and 20% experienced progression after neoadjuvant therapy. Stable disease was the predominant pattern across all disease categories. Funnel plots are depicted in Additional file 3: Figures. S2 thru S4. Although funnel plots were found to be symmetric on visual inspection, small study effects were more pronounced for stable disease (Begg’s test *p* = 1, Egger’s test *p* = 0.02) and progressive disease analyses (Begg’s test *p* = 0.135, Egger’s test *p* = 0.033).

**Fig. 4:**
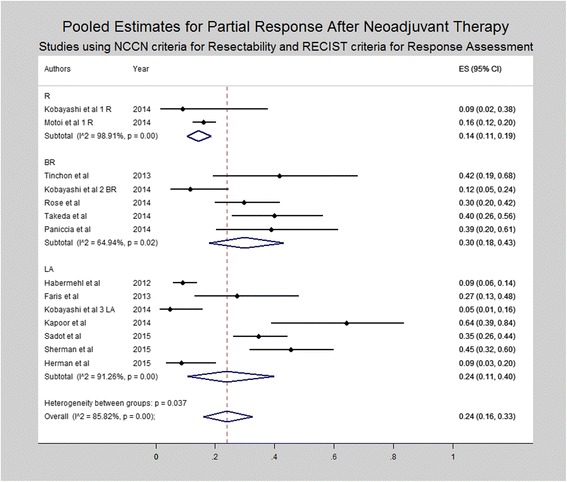
Forest plots depicting pooled estimates for partial response at the time of restaging during or after neoadjuvant therapy. Only studies utilizing NCCN or AHBPA/SSO/SSAT criteria were included in the forest plot

**Fig. 5:**
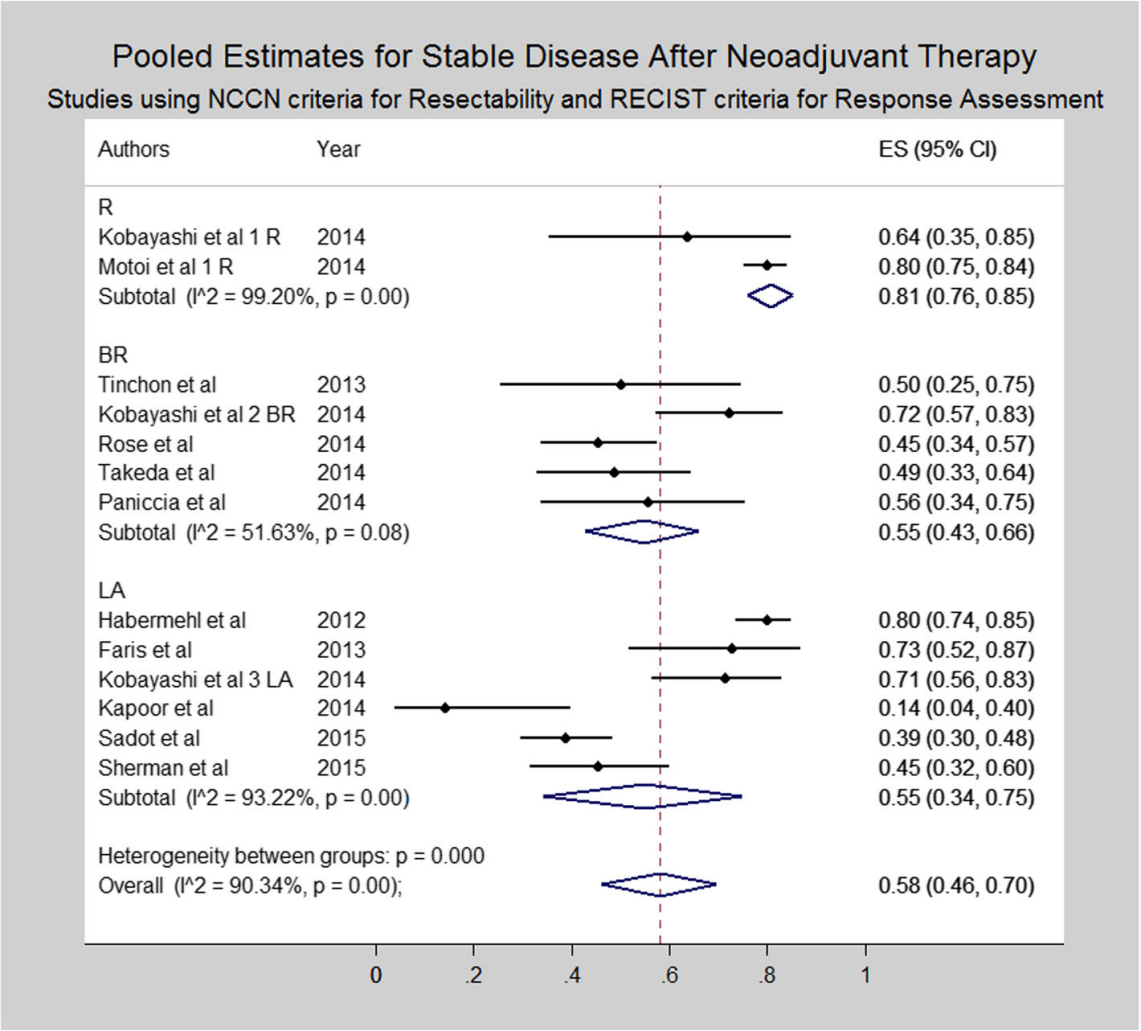
Forest plots depicting pooled estimates for stable disease at the time of restaging during or after neoadjuvant therapy. Only studies utilizing NCCN or AHBPA/SSO/SSAT criteria were included in the forest plot

**Fig. 6:**
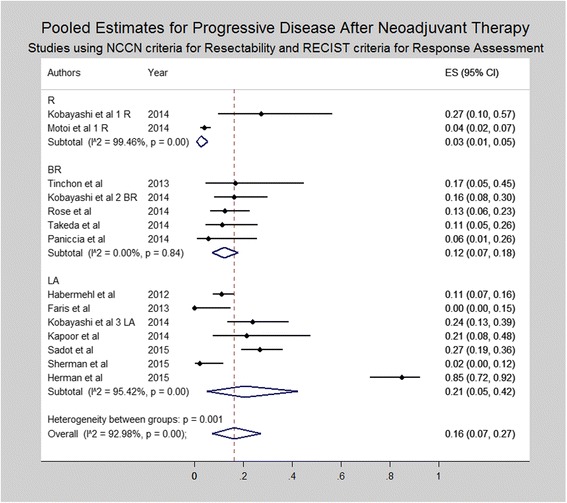
Forest plots depicting pooled estimates for progressive disease at the time of restaging during or after neoadjuvant therapy. Only studies utilizing NCCN or AHBPA/SSO/SSAT criteria were included in the forest plot

Subset analysis was performed for studies utilizing the NCCN/AHPBA/SSO/SSAT criteria for resectability and RECIST criteria for radiologic response (Figs. 4, 5, and 6). Similar to the results of all studies, subset analysis revealed an overall complete response of 0% (0–1%) including 0% (95% CI 0–0%) for resectable disease, 1% (95% CI 0–4%) for borderline resectable and 1% (95% CI 0–3%) for locally advanced disease. Similarly, rates of partial response were 14% (95% CI 11–19%) for resectable, 30% (95% CI 18% - 43%) for borderline resectable and 24% (95% CI 11–40%) for locally advanced patients. Rates of stable disease were 81% (95% CI 76–85%), 55% (95% CI 43–66%) and 55% (95% CI 34–75%) for resectable, borderline resectable, and locally advanced patients, respectively. Rates of progressive disease varied, being 3% (95% CI 1–5%) for resectable disease, 12% (95% CI 7–18%) for borderline resectable, and 21% (95% CI 5–42%) for locally advanced patients.

### Exploration and resection rates

Figure 2 provides a summary and Table 4 provides details of the surgical resection rates after neoadjuvant therapy for pancreatic adenocarcinoma. Exploration and resection rates were calculated on an intention-to-treat basis, i.e., denominator included all patients who underwent neoadjuvant therapy. These data were available for most included studies. Exploration rates varied by the disease category, i.e., resectable (86%), borderline resectable (77%) and locally advanced (32%). As expected, the highest resection rates were noted for resectable patients (76%), followed by borderline resectable (69%), and locally advanced disease (26%). R0 resection rates were 63% for resectable patients, 54% for borderline resectable, and 23% for locally advanced disease on an intention-to-treat basis. For all patients in all categories who were selected for resection after neoadjuvant therapy, 85% had R0 resection. Funnel plots are depicted in Additional file 3:Figures S5 thru S6. No significant small study effects were noted for exploration (Begg’s test *p* = 0.234, Egger’s test *p* = 0.085) and resection rates (Begg’s test *p* = 0.528, Egger’s test *p* = 0.457).

**Table 4:** Surgical exploration and resection rates after neoadjuvant therapy

Surgical treatment	Explored/*N*	Resected/*N*	R0/*N*	Resected/explored	R0/resected
Resectable	*86%*	*76%*	*63%*	*92%*	*88%*
	[79–92%]	[68–84%]	[51–75%]	[85–97%]	[80–94%]
	*I* ^2^ = 83.91%	*I* ^2^ = 86.02%	*I* ^2^ = 90.69%	*I* ^2^ = 83.37%	*I* ^2^ = 81.82%
	(*n* = 18)	(*n* = 18)	(*n* = 14)	(*n* = 18)	(*n* = 14)
Borderline resectable	*77%*	*69%*	*54%*	*91%*	*84%*
	[66–86%]	[59–78%]	[37–71%]	[84–96%]	[67–96%]
	*I* ^2^ = 87.62%	*I* ^2^ = 88.98%	*I* ^2^ = 95.15%	*I* ^2^ = 70.68%	*I* ^2^ = 93.75%
	(*n* = 15)	(*n* = 18)	(*n* = 16)	(*n* = 15)	(*n* = 16)
Locally advanced	*32%*	*26%*	*23%*	*86%*	*82%*
	[23–42%]	[19–34%]	[15–33%]	[73–95%]	[69–93%]
	*I* ^2^ = 91.44%	*I* ^2^ = 89.42%	*I* ^2^ = 91.18%	*I* ^2^ = 86.04%	*I* ^2^ = 82.75%
	(*n* = 22)	(*n* = 24)	(*n* = 18)	(*n* = 19)	(*n* = 15)
BR + LA	*65%*	*53%*	*39%*	*89%*	*85%*
	[49–79%]	[36–70%]	[27–52%]	[80–96%]	[79–90%]
	*I* ^2^ = 95.67%	*I* ^2^ = 96.04%	*I* ^2^ = 92.74%	*I* ^2^ = 85.80%	*I* ^2^ = 35.55%
	(*n* = 20)	(*n* = 22)	(*n* = 20)	(*n* = 19)	(*n* = 19)
All patients	*64%*	*55%*	*45%*	*91%*	*85%*
	[57% -71%]	[48–62%]	[38–52%]	[87–94%]	[80–89%]
	*I* ^2^ = 95.27%	*I* ^2^ = 95.34%	*I* ^2^ = 94.94%	*I* ^2^ = 84.53%	*I* ^2^ = 85.05%
	(*n* = 84)	(*n* = 90)	(*n* = 74)	(*n* = 79)	(*n* = 70)

Values in [] reflect the 95% confidence interval. *I* ^2^ is a quantitative indicator of heterogeneity among the studies, *n* refers to the number of studies reporting the outcomes

**Fig. 7:**
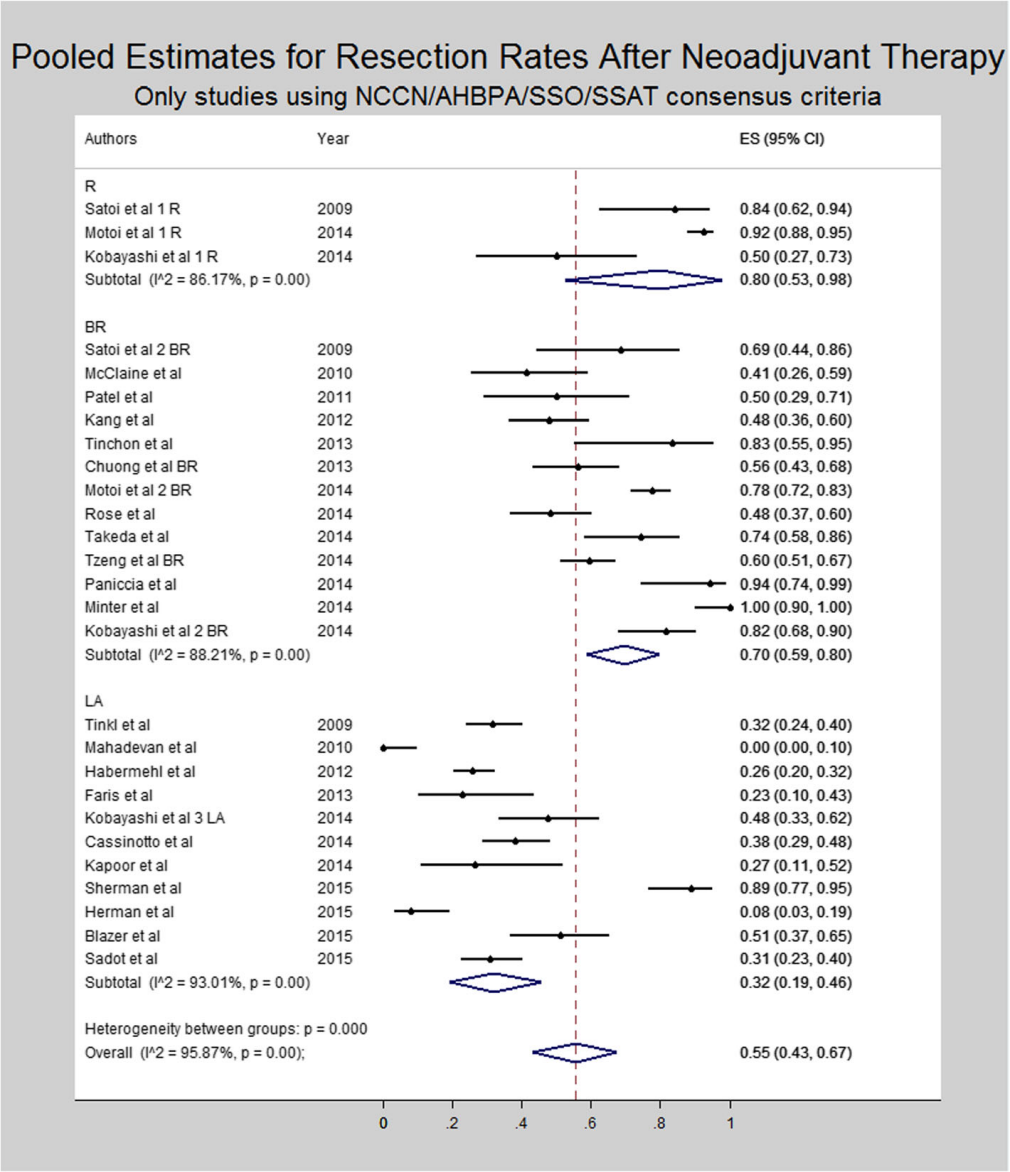
Forest plot depicting pooled estimates for resection rates after neoadjuvant therapy. Only studies utilizing NCCN or AHBPA/SSO/SSAT criteria were included in the forest plot

Subset analysis for resection rates was performed for studies utilizing the NCCN/AHPBA/SSO/SSAT criteria for resectability (Fig 7). Resection rates on an intention to treat basis were 80% for resectable patients (95% CI 53–98%), 70% for borderline resectable patients (95% CI 59–80%) and 32% for locally advanced patients (95% CI 19–46%).

### Pathologic response

Figure 2 provides a summary and Additional file 2: Table S2 provides details of the pathologic responses after neoadjuvant therapy. Pathologic grading of the tumor response was available in 36 studies. The Evans grading system was used in 16 studies. Regardless of the pathologic grading system used, if complete pathologic responses were reported, they were counted as grade 4 Evans responses and > 90% tumor destruction was considered grade 3. For further analysis we focused on the Evans grading system for response to neoadjuvant therapy. Grade 2a (37%) or 2b (27%) were the predominant histopathologic response types. Funnel plots were not generated given small number of studies. There is risk of bias with these calculations given the limitation of small number of studies which reported Evans criteria.

### Surgical morbidity and mortality

The overall morbidity was calculated for all patients undergoing exploration whereas grade 3 or higher morbidity was calculated for patients who underwent resection. Twenty eight studies reported the morbidity rates and 41 studies reported the perioperative mortality rates. Overall morbidity, grade 3 or higher morbidity and perioperative mortality rates were 39% (95% CI 33–45%), 18% (13–23%) and 1% (0–2%), respectively. Funnel plots are depicted in Additional file 3: Figures S7 and S8. No significant small study effects were noted (Begg’s test and Egger’s test > 0.05).

### Overall survival

A meta-analysis of the median OS and PFS was performed using the strategy proposed by Gillen et al. [23] Fig. 2 provides a summary and Table 5 provides details of median OS for all patients stratified by the extent of the disease and resection status. Pooled median OS in studies with single-agent chemotherapy (gemcitabine, 5-fluorouracil, or capecitabine) was 14.7 months (range 9.1–47 months). Studies with two drug regimens were noted to have a pooled median OS of 16.1 months (range 7.3–45 months). Pooled median OS was longest in the studies utilizing FOLFIRINOX with median OS of 22.1 months (range 16.7–34 months) followed by GTX (Gemcitabine, Taxane, Capecitabine) at 19.4 months (range 15.6–25 months). Funnel plots were not generated as a different methodology as previously reported by Gillen et al. was utilized.

**Table 5:** Summary of Median Overall Survivals in the various studies included in the current review

		Overall survival (months)		
		All patients	Resected	Not resected
Resectable	Median (range)	18.2 (13–28)	30.0 (24.5–46)	10 (9–11)
	*n*	9	8	5
Borderline resectable	Median (range)	19.2 (9.1–45)	27.4 (19.3–41.2)	12.9 (9–15.5)
	*n*	11	8	7
Locally advanced	Median (range)	13.6 (7.3–32.5)	18.7 (14.4–24.9)	12.6 (8–19.7)
	*n*	28	8	7
BR + LA	Median (range)	14.7 (10.6–47.2)	26 (13–47.4)	12.4 (8.8–17)
	*n*	16	11	9
All patients	Median (range)	15.3 (7.3–47.2)	24.4 (11.7–47.4)	11.5 (5.7–19.7)
	*n*	63	39	32
Progression-free survival (months)				
		All patients	Resected	Not resected
Resectable	Median (range)	8.4 (6.2–10.4)	14.9 (8.4–23)	11^*^
	*n*	2	4	1
Borderline resectable	Median (range)	9 (2.4–21.1)	15.4 (4.7–23.2)	2.1^*^
	*n*	6	6	1
Locally advanced	Median (range)	9.3 (4–17.6)	12.9 (9.6–22.5)	5.7 (4–8)
	*n*	20	6	3
BR + LA	Median (range)	9.9 (6.5–27.4)	12.5 (9–19.7)	7.5 (7.1–7.6)
	*n*	9	4	2
All patients	Median (range)	9.3 (2.4–27.4)	12.9 (4.7–23.2)	5.8 (2.1–11)
	*n*	31	20	7

^*^Only one observation. *n* number of studies reporting the outcome. Values in parenthesis indicate ranges

## Discussion

With the expert consensus statement guidelines released in 2009, providing a uniform foundation was created for categorizing non-metastatic pancreatic adenocarcinoma into resectable, borderline resectable, and locally advanced disease. Given the overall poor prognosis of pancreatic adenocarcinoma, neoadjuvant therapy is being increasingly studied in the treatment of pancreatic adenocarcinoma across all anatomic categories including resectable, borderline resectable, and locally advanced disease. In recent years, we have seen several advancements with the introduction of newer chemotherapeutic regimens (FOLFIRNOX and gemcitabine-nab-paclitaxel) and their integration into the neoadjuvant therapeutic paradigm.

The current review was undertaken to summarize the outcomes for neoadjuvant therapy in pancreatic adenocarcinoma post-2009 which marks the release of the consensus statements as well as the introduction of newer neoadjuvant regimens. Integrating the breakdown of pancreatic adenocarcinoma by anatomic category with chemotherapy and surgical characteristics, the current analyses includes 5520 patients from 96 studies. The resulting data formulate a perspective on critical outcomes including toxicities, responses, surgical, and survival characteristics that can help guide patients and physicians.

One concern of neoadjuvant therapy is that possible toxicities might contribute to patient morbidity or mortality. Grade ≥ 3 toxicities were seen in 36% of patients with a higher incidence of grade ≥ 3 hematologic (25%) than non-hematologic toxicity (16%); however, mortality during neoadjuvant therapy was low (95% CI 0–1%). The hospitalization rate during neoadjuvant therapy was 21%. Our data suggest that these toxicities were manageable since majority of patients (91%) were able to complete neoadjuvant therapy as reported in 42 studies (not considering dose modifications).

Neoadjuvant therapy remains most controversial for patients with resectable pancreatic adenocarcinoma. Critics of the neoadjuvant approach have cited disease progression, patient anxiety, and lack of proven benefit of neoadjuvant therapy in patients with resectable disease, whereas the proponents propose that neoadjuvant therapy allows testing of tumor biology and spares unnecessary surgery in patients who are destined to develop rapidly progressive metastatic disease in the immediate post-operative period. Stable disease remains the predominant radiologic response after neoadjuvant therapy. Progression was seen in a minority of patients (16%) overall and in an even smaller proportion of patients with resectable disease (11%). Surgical morbidity and mortality after neoadjuvant therapy remain acceptable with grade 3 or higher morbidity of 18% and mortality of 1%. These are comparable to morbidity and mortality after upfront resection. These data suggest that progression during neoadjuvant therapy is rare and neoadjuvant therapy does not increase morbidity or mortality after surgery.

Although the main goal of any neoadjuvant strategy is to prolong survival, another goal is to improve R0 resection, as positive resection margins have been shown to be associated with worse survival [126–128]. Previous studies of adjuvant therapy have reported an R1 resection rate of 24–42% [126, 127, 129, 130] whereas margin positive rate has been < 10% in previous trials of neoadjuvant therapy in selected centers [131, 132]. In the current study, for patients (all disease categories) who were explored after neoadjuvant therapy, over 80% of the patients had R0 resection. Importantly, patients with resectable disease undergoing resection after the receipt of neoadjuvant therapy have high likelihood of margin negative resection with an improved estimated median survival of up to 30 months. Additionally, one could argue that R0 resection rate of 63% on an intention to treat basis may seem low—this is in par with previous surgical series reporting an R1 resection rate of >30% including ESPAC 3 (R1 rate = 35%) [126, 129, 130]. These data suggest that neoadjuvant therapy increases the likelihood of R0 resection among all anatomic subcategories of pancreatic adenocarcinoma.

In the current review, median OS for all patients with resectable disease who underwent neoadjuvant therapy was 18 months. Previous trials of adjuvant therapy have reported a median OS of approximately 20–23 months [129, 130, 133, 134]. However, the results are not comparable as the adjuvant therapy trials only included patients who were able to undergo resection, recovered well after surgery to undergo chemotherapy, and in some studies were required to not have metastatic disease on a postoperative scan [130]. Previous studies have suggested that only approximately 50–60% of the patients are able to receive adjuvant therapy after surgery [16–18]. In the current study, resectable patients who were able to undergo resection after neoadjuvant therapy had a median OS of up to 30 months. Median survival among those patients with resectable tumors who were unable to undergo resection for various reasons was only 10 months. It can be speculated that these patients might not have benefitted from upfront resection and might have developed rapidly progressive disease in the postoperative period. Conversely, NAT could have precluded these patients from undergoing a curative resection. Although adjuvant therapy can add some incremental prolongation of survival, surgery remains the only curative-intent treatment option. At this time upfront surgery followed by a combination of gemcitabine and capecitabine remains the standard of care for resectable pancreatic adenocarcinoma based on the results of ESPAC-4 trial [135]. In this phase 3, open label, randomized controlled trial median OS of patients in the gemcitabine plus capecitabine arm was 28 months compared to 25.5 months for gemcitabine alone (HR 0.82, 95% CI 0.68–0.98, *p* = 0.032. Neoadjuvant therapy for resectable pancreatic adenocarcinoma remains an area of active investigation. One of the ongoing trials S1505 conducted by Southwest Oncology Group (A randomized phase II study of perioperative mFOLFIRINOX vs. gemcitabine/nab-paclitaxel as therapy for resectable pancreatic adenocarcinoma, clinicaltrials.gov NCT02562716) is evaluating the role of newer multidrug regimens in neoadjuvant setting, in patients with resectable pancreatic adenocarcinoma, with a primary endpoint of OS. Such studies will shed further light on the effectiveness of neoadjuvant therapy in resectable pancreatic adenocarcinoma [136]. Additionally, all attempts should be made to study NAT in resectable pancreatic cancer in the context of clinical studies.

The role of neoadjuvant therapy in patients with borderline resectable pancreatic adenocarcinoma is well recognized [7]. Interestingly, for patients with locally advanced disease, 22% were eventually able to achieve an R0 resection in the current review. R0 resection rates were 82% in patients who underwent resection. A recent meta-analysis based on FOLFIRINOX for locally advanced pancreatic cancer reported a similar resection rate of 25.9% and R0 resection rate of 78.4% [137]. As more effective chemotherapeutic regimens become available, more patients may be able to undergo resection and may experience improvement in OS. One such study (clinicaltrials.gov NCT 02839343, Alliance 021501) is investigating the role of pre-operative extended chemotherapy (mFOLFIRINOX pre-operatively and FOLFOX post-operatively) with or without hypofractionated radiation therapy in patients with borderline resectable pancreatic adenocarcinoma.

Our study has several limitations given the heterogeneity among studies, heterogeneity in the regimens, use of radiation in some studies, and varying duration and tolerability of treatments—however, this is an expected and inherent feature of any systematic review. Heterogeneity between the patient populations, chemotherapy regimens, etc. could account for wide confidence intervals for some of the reported outcomes. The lack of standardization in the surgical techniques and pathologic assessment of specimens also makes comparison across studies challenging. Ideally, a meta-analysis should be performed using individual patient data; however, individual patient data may not always be available or practical. Although meta-analysis may be superior to individual studies, its qualitative results need to be viewed carefully even when the analysis is performed on data quantitatively larger than single-institutional studies. An extensive forward and backward search was undertaken to ensure the completeness of literature along with abstracts of selected meetings. However, there is a possibility that some studies might have been missed.

In addition, although the consensus guidelines attempt to separate patients into three distinct categories (R, BR and LA), in practice there can be some overlap between the BR and LA categories. At the extremes of BR and LA, the distinction is well-characterized, but there are several patients where the distinction is not that obvious (for example patients with approximately 180 degree involvement of SMA). Thus, some of the comparisons in the studies between the different categories need to be interpreted with caution. It also needs to be borne in mind that the adoption of FOLFIRINOX and gemcitabine-nab paclitaxel regimens is relatively recent (2011 and 2013) with lack of robust randomized controlled data. Our study used the Evans criteria for pooling the estimates of pathologic response [138]. Although Evans criteria remain the most widely used pathologic criteria for response assessment, these are not universally adopted [139]. Given the limitations of the current study, we could not perform a head to head comparison of morbidity and mortality of surgery followed by adjuvant therapy vs neoadjuvant therapy followed by surgery. Finally, the radiologic response rates may vary with the use of radiation therapy and also type of radiation therapy, i.e., IMRT vs SBRT. Response after chemoradiation may have different biological relevance than response after systemic chemotherapy alone. We could not decipher the effect of radiation given the differences in the duration, doses, and type of radiation treatments. In spite of its limitations, the current study provides a comprehensive summary of the data on neoadjuvant therapy for pancreatic adenocarcinoma and these data can be used for counseling at the time of informed consent for those patients interested in neoadjuvant therapy.

## Conclusions

In conclusion, the current study reviewed data on 5520 patients with pancreatic adenocarcinoma (all stages of all non-metastatic disease: resectable, borderline resectable, and locally advanced) treated with neoadjuvant therapy and published in the last 7 years. The data summarize the surgical treatment, radiologic response, toxicity, pathologic response, morbidity and mortality as well survival of patients with pancreatic adenocarcinoma treated with neoadjuvant therapy. Although surgery first continues to be the standard of care and the only curative-intent treatment option for resectable disease, the results of this systematic review demonstrate increasing adoption of neoadjuvant therapy with some favorable outcomes. The introduction of more effective chemotherapeutic agents will help in increasing the acceptance of neoadjuvant therapy in certain subpopulations. Ongoing therapeutic/clinical trials to test, validate and introduce newer and more effective chemotherapeutic agents will go a long way in tackling this lethal disease for which we may have reached the peak of our surgical expertise.

## Additional files

Additional file 1: Table S1. Summary of studies included in current meta-analysis (DOCX 54 kb)

Additional file 2: Table S2. Pathologic response to neoadjuvant therapy (DOCX 17 kb)

Additional file 3: Funnel plots for assessment of publication bias and small study (PPTX 88 kb)
