# Supplementary material for: Neoadjuvant treatment of pancreatic adenocarcinoma: a systematic review and meta-analysis of 5520 patients
Source: World J Surg Oncol. 2017 Oct 10;15:183. doi: 10.1186/s12957-017-1240-2 (PMC5634869; doi:10.1186/s12957-017-1240-2)
Supplement: Supplementary file 1 — Summary of studies included in current meta-analysis (DOCX 54 kb) [file 12957_2017_1240_MOESM1_ESM.docx]

| Additional file 1: Table 1 Summary of studies included in current meta-analysis | | | | | | | | | | | | | | | | | | | |
| --- | --- | --- | --- | --- | --- | --- | --- | --- | --- | --- | --- | --- | --- | --- | --- | --- | --- | --- | --- |
| Serial No | Authors | Article type | Journal | Year of publication | Type of study | Institution | Study duration | Chemo type | RT | Vascular resection | Age | Gender | Anatomic criteria | Radiologic criteria | Cycles of chemo | RT dose | Toxicity criteria | Adjuvant therapy | Follow up |
| 1 | Alline et al | ASCO 2015 Abstract # 421 | Journal of Clinical Oncology | 2015 | Retrospective | Montpellier University Hospital, France | 2009 - 2013 | FOLFIRINOX | Yes | 6/13 (46%) | Median 60 (40 -74) | Male 17/31 (54.8%), Female (14/31 (45.2%) | NCCN guidelines | NA | Median 4 | Not mentioned | grading criteria not mentioned | Not mentioned |  |
| 2 | Alagappan et al | ASCO 2015 Abstract # 433 | Journal of Clinical Oncology | 2015 | Retrospective | Stanford University, Stanford | 2002 - 2014 | RT alone | Yes | - | Median 75.2 | Male 109/208 (52.4%), 99/208 (47.6%) | Not mentioned |  |  |  |  |  |  |
| 3 | Barbour et al | ASCO 2015 Abstract # 387 | Journal of Clinical Oncology | 2015 | Phase II | Multi-institutional, Australia | Not mentioned | Gem + Nab paclitaxel | No | None | Median 63 (43 - 79) | Male 17/41 (41%), Female 24/41 (59%) | Similar to MD Anderson criteria | NA | 6 | NA | grading criteria not mentioned | 18/30 (60%) |  |
| 4 | Blazer et al | Original | Annals of Surgical Oncology | 2015 | Retrospective | The Ohio State University, Columbus | January 2011 - August 2013 | FOLFIRINOX | Yes | 4/22 (18.2%) | Median 62.4 (40 -81) | Male 23/43 (53.5%) | AHPBA/SSO/SSAT consensus criteria | Not mentioned | Mean 4.9 | 36 Gy | NCI CTCAE v4 | 17/22 (77%) | Median 13.3 months |
| 5 | Carvalho et al | ASCO 2015 Abstract # 458 | Journal of Clinical Oncology | 2015 | Retrospective | Centro Oncologico Antonio Erminio de Moraes, Sao Paulo, Brazil | - | FOLFIRINOX | Yes | - | Median 61 (38 - 78) | Male 6/14 (43%), Female 8/14 (57%) | Not mentioned |  | Median 11 | 55 Gy |  |  | Median 11.7 months |
| 6 | Chllamma et al | ASCO 2015 Abstract # 417 | Journal of Clinical Oncology | 2015 | Retrospective | Princess Margaret Hospital, Toronto | December 2011 - April 2014 | FOLFIRINOX | No | - | Median 64 (28 - 76) | Male 57 ( | Not mentioned |  | Median 6 | NA | Not mentioned separately for LA patients | Not mentioned |  |
| 7 | Ferrone et al | Original | Ann Surg | 2015 | Retrospective | Massachusetts General Hospital, Boston | April 2011- March 2014 | FOLFIRINOX | Yes | - | Median 62, range (38-77) | Male 21 (52.5%) | AHPBA/SSO/SSAT consensus | Not mentioned. Review by tumor board | Median 8 (range 1-24) | 50.4 Gy | NA |  | Median 13.5 months |
| 8 | Golcher et al | Original | Strahlenther Onkol | 2015 | Phase II study | Multi-institutional, Germany | June 2003 - December 2009 | Gem + Cis | Yes | - | Median 62.5 range (33 - 76) | Male 18 (55%) Female 15 (55%) | vascular involvement < = 180° of one of the  peripancreatic major vessels (portal vein, confluent of superior mesenteric vein and splenic vein, superior mesenteric artery, celiac trunk with its major branches splenic artery and hepatic artery, superior mesenteric vein) (criteria according to Lu et al., 1997). | RECIST | Chemo on day 1, 8, 22, 29 per protocol | Median 55.8 Gy (range 45 - 57.6 Gy) | NCI CTCAE v 2, RTOG/EORTC |  | Median 61 months - alive patients |
| 9 | Hajj et al | ASCO 2015 Abstract # 377 | Journal of Clinical Oncology | 2015 | Retrospective | Memorial Sloan Kettering Cancer Center, New York | November 2006 - November 2012 | Multiple | Yes | - | Mean 64.1 (range 36.7 - 84.1) | Male 71/134 (53%), Female 63/134 (47%) | Other - T4 disease or unreconstructible involvement of portal vein or hepatic artery or unresectable on exploration | Not mentioned | Not mentioned | Not mentioned | grading criteria not mentioned | 15/26 (58%) | Median follow up 20.1 months |
| 10 | He et al 1 Chemo + CRT | ASCO 2015 Abstract # 341 | Journal of Clinical Oncology | 2015 | Retrospective | Johns Hopkins Medical Institutions, Baltimore | 2011 - 2014 | Not mentioned | Yes | 16/43 (37%) | Median 61 (iqr 11) | Male 20/43 (47%) Female 23/42 (53%) | Not mentioned | NA | Not mentioned | 33 Gy | NA | Not mentioned |  |
|  | He et al 2 CRT |  |  |  |  |  |  |  | Yes | 11/82 (13%) | Median 63 (iqr 13) | Male 50/82 (61%), Female 42/82 (39%) | Not mentioned | NA | Not mentioned | Not mentioned | NA | Not mentioned |  |
|  | He et al 3 Chemo |  |  |  |  |  |  |  | No | 8/26 (31%) | Median 65 (iqr 10) | Male 16/26 (61%), Female 10/26 (39%) | Not mentioned | NA | Not mentioned | NA | NA | Not mentioned |  |
| 11 | Herman et al | Original | Cancer | 2015 | Phase II | Multi-institutional, US | 2010-2012 | Gem | Yes | - | Median 67 (range 35 -87) | Male 31/49 (63%), Female 18/49 (37%) | AHPBA/SSO/SSAT consensus criteria | RECIST | 7 | 33 Gy | NCI CTCAE v4, RTOG | Not mentioned |  |
| 12 | Marthey et al | Original | Annals of Surgical Oncology | 2015 | Prospective observational | Multi-institutional, France | February 2010 - February 2012 | FOLFIRINOX | Yes | - | Median 61 (range 37 - 79) | Male 46/77 (60), Female 31/77 (40) | Other multidisciplinary review - at least 180 degree contact or encasement of major arterial or venous structures | RECIST | Median 5 | 54 Gy | NCI CTCAE v3 |  | Median 15 months |
| 13 | Rashid et al | ASCO 2015 Abstract # 374 | Journal of Clinical Oncology | 2015 | Retrospective | H Lee Moffitt Cancer Center, Tampa | January 2006 - December 2013 | GTX | Yes | - |  |  |  |  |  |  |  |  |  |
| 14 | Sadot et al | Original | Annals of Surgical Oncology | 2015 | Retrospective | Memorial Sloan-Kettering Cancer Center, New York | July 2010 - October 2013 | FOLFIRINOX | Yes | 8/31 (28%) | Median 64 (range 37 - 81) | Male 52/101 (52%) | NCCN criteria | RECIST 1.1 | Median 6 | Not mentioned | NCI CTCAE v4 | Not mentioned | Median 12 months |
| 15 | Sherman et al | Original | Cancer | 2015 | Prospective | Columbia University, New York | February 2010 - December 2013 | GTX | Yes | - | Range 44- 83 | Male 19/45, Female 26/45 | Similar to AHPBA/SSO/SSAT consensus. > 180 degree arterial involvement or extensive venous involvement | RECIST | 6 per protocol | 50.4 Gy | Specific grading criteria not mentioned |  |  |
| 16 | Tomimaru et al | Original | Japanese J Clin Oncol | 2015 | Retrospective | Osaka University, Osaka, Japan | 2007 - 2013 | Gem | Yes | - | Mean 73 +/- 4 | Male 7, Female 5 | If tumor did not involve hepatic artery, celiac trunk or SMA. Venous involvement of PV/SMV if vessel proximal and distal to area of involvement suitable for reconstruction available for reconstruction | Not mentioned | 1 cycle (Day 1, 8, 15 of 28 day cycle) | 40 or 50.4 Gy | NCI CTCAE V4 | Yes (6 cycles of gem) | Not mentioned |
| 17 | Wang-Gillam et al 1 Arm A | ASCO 2015 Abstract # 338 | Journal of Clinical Oncology | 2015 | Phase 1b | Washington University School of Medicine, St Louis | Not mentioned | FOLFIRINOX | No | - | Median 67.5 (55 - 75) | Male 2/6 (33%) Female 4/6 (67%) | Not mentioned | RECIST | Median 3 | NA |  |  |  |
|  | Wang-Gillam et al 2 Arm B | ASCO 2015 Abstract # 338 | Journal of Clinical Oncology | 2015 | Phase 1b |  |  | FOLFIRINOX + PF-04136309 | No | - | Median 62 (45 - 73) | Male 17/39 (43.6%), Female 22/39 (56.4%) | Not mentioned | RECIST | Median 6 | NA |  |  |  |
| 18 | Cassinotto et al | Original | Radiology | 2014 | Prospective | Universitaire de Bordeaux, France | June 2009 - May 2013 | FOLFIRINOX, GEMOX | Yes | 25 | Mean 61.7 +/- 9 | Male 32(68%) Female 15 | AHPBA/SSO/SSAT consensus | Not mentioned | 4 cycles | 60 Gy | NA | Not mentioned |  |
| 19 | Chakraborty et al | Original | Gastrointestinal Cancer Research | 2014 | Phase II study | University of Virginia, Charlottesville | September 2010 - February 2012 | Cape | Yes | - | Median 66 (range 51 - 82) | Male 5 (38%), Female 8 (62%) | MD Anderson criteria | RECIST 1.1 | 1 Cycle concurrent with radiation | 50 Gy | NCI CTCAE V4 | 2/5 resected received adjuvant gemcitabine |  |
| 20 | Chao et al | Original | BMC Surgery | 2014 | Retrospective | National Cheng Kung University Hospital, Tainan, Taiwan | May 2003 - December 2009 | GEMOX | Yes | - | Median 63.5 (range 39 - 80) | Male 27, Female 14 | 1) Abutment or encasement of celiac or SMA 2) Involvement of portal vein at the confluence of splenic and SMV 3) Severe extra pancreatic soft tissue involvement | RECIST | Multiple regimens | 50.4Gy | NA | Not mentioned | Not mentioned |
| 21 | Chen et al | Original | Annals of Surgical Oncology | 2014 | Retrospective | Fox Chase Cancer Center, Philadelphia | 2000 - 2014 | Multiple | Yes | - | Range 38 - 82) | Male 37 | NCCN guidelines | NA | Multiple, maximum of 6 cycles | 50 - 56 Gy | NA | Yes (subset of patients) |  |
| 22 | Christians et al | Original | The Oncologist | 2014 | Retrospective | Medical College of Wisconsin, Milwaukee | July 2010 - December 2012 | FOLFIRINOX | Yes | 10/12 resected patients | Mean 60.6 | Male 9, Female 9 | Medical College of Wisconsin clinical/radiographic staging - similar to MD Anderson criteria | Not mentioned | Mean 4.3, sd 1.6 | 50.4 Gy | Criteria not mentioned | 1/12 resected patients |  |
| 23 | Combs et al | Original | Annals of Surgical Oncology | 2014 | Retrospective | University Hospital of Heidelberg, Heidelberg, Germany | 2004 - 2012 | Gem | Yes | - | Median 66 (range 42 - 95) | Male 128 (44%), Female 161 (56%) | 1/ extension beyond 180 degree of SMA, infiltration of celiac trunk, occlusion of SMV or portal vein, aortic invasion | NA | Not mentioned | 50.4 - 50 Gy | NA | Not mentioned |  |
| 24 | Cuneo et al | Original | Translational Oncology | 2014 | Pilot study/Prospective | University of Michigan, Ann Arbor | October 2008 - December 2009 | GEMOX | Yes | N/A | Mean 60.7, median 59 (range 50 -71) | Not mentioned | Not mentioned | NA | 1 cycle concurrent with radiation (Gem on day 1, 8 and 15, Ox on day 1 and 15) | 30 Gy | NA | Not mentioned |  |
| 25 | Eguchi et al | Original | Cancer Chemother Pharmacol | 2014 | Phase 1 | Osaka University, Japan | December 2009 - December 2012 | Gem + S1 | Yes | - | Median 66(56 - 79) | Male 15/21 (71.5%), Female 6/21 (28.5%) | Other - No involvement of HA, celiac trunk or SMA, no metastases, venous involvement of SMV/PV with suitable vessel proximal and distal to the area of resection, if tumor could be removed by distal pancreatectomy with celiac axis resection | RECIST | Concurrent with radiation | 50.4 Gy | NCI CTCAE v3 | Not mentioned |  |
| 26 | Epelboym et al | Original | World J Surg | 2014 | Retrospective | Columbia University, New York | March 1992 - December 2011 | GTX | Yes | 92/143 (64.3%) | Mean 63.5 (sd 9.9( | Male 71 (49.7%) | NCCN guidelines | Not mentioned. Retrospective | All 6 cycles preoperatively | Not mentioned | NA | Not mentioned |  |
| 27 | Esnaola et al | Original | Inernational Journal of Radiation Oncology | 2014 | Phase II study | Medical University of South Carolina, Charleston | March 2006 - November 2008 | GEMOX + Cetuximab | Yes | 5/11 | Median 60 (range 28 - 78) | Male 20 (54%), Female 17 (46%) | NCCN guidelines | RECIST | Approximately 6 cycles | 99.9 Gy | NCI CTCAE v 3 | Not mentioned | Median follow up of 46.3 months |
| 28 | Gulati et al | ASCO 2014 abstract # 274 | Journal of Clinical Oncology | 2014 | Phase II | Columbia University, New York | Not mentioned | GTX | Yes | - | Median 61 | Male 16/35 (46%), Female 19/35 (54%) | Other: localized to the pancreas, small bowel, stomach and/or encasing at least 2 vessels such as the SMA, CA, HA, PV, or SMV | RECIST | 3 | 50.4 Gy | grading criteria not mentioned | Not mentioned |  |
| 29 | Hong et al | Original | International Journal of Radiation Oncology | 2014 | Phase 1/2 study | Harvard Medical School, Boston | Protocol started in 2007 | Cape | Yes | - | Median 65 (range 49- 62) | Male 27 (54%), Female 23 (46%) | Not mentioned | Not mentioned | Capecitabine for 2 weeks during radiation | 25 Gy | Not mentioned | Yes (31/37 - adjuvant gemcitabine) | Median follow up 38 months among alive patients |
| 30 | Ioka et al | ASCO 2014 abstract # 261 | Journal of Clinical Oncology | 2014 | Phase 1 | Osaka Medical Center, Osaka, Japan | February 2006 - March 2007 | Gem + S1 | Yes | - | Range 20-79 | Not mentioned | Other: deemed unresectable by multiple surgeons, celiac artery and/or more than 50% of SMA | Not mentioned | Concurrent with radiation | 50.4 Gy | NCI CTCAE v3 | Not mentioned |  |
| 31 | James et al | ASCO 2014 abstract *# 256* | Journal of Clinical Oncology | 2014 | Interim analysis Phase II study | Yale Cancer Center, New Haven | November 2011 - August 2013 | FOLFIRINOX | No | - | Median 63 (46 - 86) | Male 18/27 (67%), Female 9/27 (33%) | Not mentioned | RECIST 1.1 | Median 7 | NA | grading criteria not mentioned | Not mentioned |  |
| 32 | Jensen et al | Original | HPB | 2014 | Phase II study | University of Minnesota Medical Center, Minneapolis | January 2005 - October 2010 | 5FU + Cisplatin + IFN | No | 1/7 (14.3%) | Mean 58.6, sd 9.01 | Male 17/23 (73.9%), Female 6/23 (26.1%) | Similar to AHPBA/SSO/SSAT criteria | Not mentioned | 1-3 cycles | 50.4 Gy | NCI CTCAE v3 | Not mentioned |  |
| 33 | Kapoor et al | Original | Indian Journal of Cancer | 2014 | Prospective pilot study | Regional Cancer Center, Chandigarh, India | January 2009 - June 2011 | Cape | Yes | - | Median 63.4 (range 48 - 72) | Male 8, Female 7 | Similar to NCCN - encasement of the celiac or SMA | RECIST | Capecitabine during radiation treatment | 30 Gy | NCI CTCAE v3 | Yes 3/4 resected patients) | Median 7.5 months |
| 34 | Kharofa et al | Original | Radiotherapy and Oncology | 2014 | Retrospective | University of Cincinnati, Cincinnati | January 2009 - November 2011 | Multiple | Yes | - | R - Median 68 (37-82). BR - Median 64 (45 - 87) | R - Male 13/30 (43%). BR - 18/39 (46%) | Medical College of Wisconsin clinical/radiographic staging - similar to MD Anderson criteria | Not mentioned | Variable | 50.4 Gy | NCI CTCAE v4 | Adjuvant therapy was considered. Number not mentioned |  |
| 35 | Kobayashi et al 1 R | Original | Pancreas | 2014 | Retrospective | Mie University School of Medicine, Mie, Japan | February 2005 - October 2010 | Gem | Yes | 2/7 (28.6%) | Mean 66.4, sd 9.9 | Not mentioned | NCCN guidelines | RECIST | 1 cycle (Day 1, 8, 22 and 29 of 29 day cycle) | 45 Gy | grading criteria not mentioned | 3/6 | Mentioned |
|  | Kobayashi et al 2 BR |  |  |  |  |  |  |  | Yes | 32/26 (88.9%) | Mean 68.8, sd 9.1 | Not mentioned | NCCN guidelines | RECIST | 1 cycle (Day 1, 8, 22 and 29 of 29 day cycle) | 45 Gy | grading criteria not mentioned | 26/36 | Mentioned |
|  | Kobayashi et al 3 LA |  |  |  |  |  |  |  | Yes | 19/20 (95%) | Mean 66.1, sd8.8 | Not mentioned | NCCN guidelines | RECIST | 1 cycle (Day 1, 8, 22 and 29 of 29 day cycle) | 45 Gy | grading criteria not mentioned | 15/20 | Mentioned |
| 36 | Minter et al | ASCO 2014 abstract # 288 | Journal of Clinical Oncology | 2014 | Retrospective | University of Michigan, Ann Arbor | 2001 - 2011 | Unknown | Yes | - | Not mentioned | Not mentioned | NCCN guidelines | NA | Not mentioned | Not mentioned | NA | Not mentioned | Median 26 months |
| 37 | Mizuma et al | ASCO 2014 abstract # 283 | Journal of Clinical Oncology | 2014 | Phase II single arm study | Multi-institutional, Japan | 2008 - 2010 | Gem + S1 | No | - | Median 65 (47 - 77) | Male 20/35 (57%), Female 15/35 (43%) | Not mentioned | Not mentioned | 2 | NA | grading criteria not mentioned | Not mentioned |  |
| 38 | Motoi et al 1 R | Original | J of Hepatobiliary Pancreat Sci | 2014 | Prospective | Multi-institutional, Japan | January 2007 - December 2009 | Multiple | Yes | 41 (23.9) | Median for all patients68 (27 - 90) | Not mentioned for neoadjuvant group | NCCN guidelines | RECIST | Variable | 50 Gy | NCI CTCAE v3 | Not mentioned |  |
|  | Motoi et al 2 BR | Original | J of Hepatobiliary Pancreat Sci | 2014 | Prospective |  |  |  | Yes | 122 (77.2) | Median for all patients 65 (32 -87) | Not mentioned for neoadjuvant group | NCCN guidelines | RECIST | Variable | 45 Gy | NCI CTCAE v3 | Not mentioned |  |
| 39 | O'reilly et al | Original | Annals of Surgery | 2014 | Phase II | Memorial Sloan-Kettering Cancer Center, New York | July 2007 - December 2011 | GemOx | No | - | Median 73 (range 42 - 91) | Male 23/38 (61%), Female 15/38 (39%) | Resectable - No distant metastases, a clear fat plane between celiac and SMA, patent SMV and PV without any primary tumor involvement, no encasement of SMA or HA, no encasement of SMV or PV, no extra regional nodal disease | RECIST | 4 | NA | NCI CTCAE v3 | 26/27 (96%) | Median 39.6 months |
| 40 | Paniccia et al | Original | Medicine | 2014 | Retrospective | University of Colorado Anschutz Medical Campus, Aurora | August 2011 - September 2013 | FOLFIRINOX | Yes | 9/17 (52.9%) | Median 65 (range 58 - 69) | Male 10 (55.6%), Female 8 (44.4%) | NCCN guidelines | RECIST | Mostly 3 - 5 cycles | Not mentioned | NCI CTCAE v4 | 16/18 (88.9%) |  |
| 41 | Rose et al | Original | Annals of Surgical Oncology | 2014 | Retrospective | Virginia Mason Medical Center, Seattle | 2008 - 2012 | Gem + Docetaxel | No | 15/31 (48%) | Median 66 (IQR 61 - 73) | Male 35 (55) | AHPBA/SSO/SSAT consensus | RECIST | 8 cycles | 50.4 Gy (only 2 patients) | NA | 28/31 (90% of resected) | 21.4 months |
| 42 | Sahora et al | Original | Anticancer Research | 2014 | Phase II study | Medical University of Vienna, Vienna, Austria | August 2006 - August 2009 | Gem + Bevacizumab | No | 3/11 (27.3%) | Median 62 (range 43-80) | Male 12 (43%) Female 18 (57%) | AHPBA/SSO/SSAT consensus | RECIST 1.0 | 4 cycles (28 day each) | NA | NCI CTCAE v4 | Not mentioned | 36 months |
| 43 | Takeda et al | Original | Japanese J Clin Oncol | 2014 | Phase 1/2 | Osaka University, Osaka, Japan | January 2002 - December 2006 | Gem | Yes | 19/26 | Median 71 (range 43 - 79) | Male 23, Female12 | Similar to AHPBA/SSO/SSAT consensus. BR - portal or SMV invasion allowing for safe resection and reconstruction, celiac or SMA abutment < 180 degree | RECIST | 1 cycle during RT | 30 - 36 Gy | NCI CTCAE v2 | Not mentioned | Median 20.2 months |
| 44 | Tzeng et al | Original | HPB | 2014 | Retrospective | MD Anderson Cancer Center, Houston | 2001 - 2010 | Gem or 5FU | Yes | - | Median 63 (range 34- 81) | Female 74/141 (52.5%) | AHPBA/SSO/SSAT consensus or MD Anderson criteria | NA | Not mentioned | Not mentioned | NA | Not mentioned |  |
| 45 | Tzeng et al | Original | Journal of Gastrointestinal Surgery | 2014 | Retrospective | MD Anderson Cancer Center, Houston | 2002 - 2007 | Gem + Cis | Yes | 26/95 (27.4%) | Median 65.5 (range 38 - 79) | Female 54 (47%) | MD Anderson criteria | NA | Variable | 30 Gy or 50.5 Gy | NA | 9/95 (9.5%) |  |
| 46 | Wo et al | Original | Radiotherapy and Oncology | 2014 | Phase I study | Harvard Medical School, Boston | December 2009 - August 2011 | Cape | Yes | - | Mean 62.6 (range 57 - 75) | Female 6/10 | Not mentioned | NA |  |  | Not mentioned | Not mentioned |  |
| 47 | Alvarez et al | Original | British Journal of Cancer | 2013 | Prospective | Spanish National Cancer Research Center, Madrid, Spain | - | Gem + Nab paclitaxel | No | - | Median 58 (range 41 - 80) | Male 10/16, Female 6/16 | Not mentioned | RECIST 1.1 | 2 cycles | NA | NCI CTCAE v3 | Not mentioned |  |
| 48 | Araujo et al | Original | HPB | 2013 | Retrospective | Memorial Sloan-Kettering Cancer Center, New York | September 2001 - July 2011 | Gem | Yes | 4/29 (14%) | Median 64 (iqr 61-72) | Male 14 (48%) | MD Anderson criteria | NA | variable, 2 months median | Median 50.4 Gy | NCI CTCAE v4 | Not mentioned |  |
| 49 | Boone et al | Original | Journal of Surgical Oncology | 2013 | Retrospective | University of Pittsburgh Medical Center, Pittsburgh | February 2011 - October 2012 | FOLFIRINOX | Yes | 5/9 (56%) | Median 59 (range 42 - 73) | Male 12 (48%), Female 13 (52%) | AHPBA/SSO/SSAT consensus | NA | Mean 6 cycles | 36 Gy | NCI CTCAE | Not mentioned |  |
| 50 | Cetin et al | Original | Gastrointestinal Cancer Research | 2013 | Phase II study | University of Massachusetts Medical School, Worcester | June 2006 - March 2011 | Gem + Cetuximab | Yes | - | Mean 63.5, median 66, range 48 - 78 | Male 7/11 (63.6%) | Other criteria - Tumor size > 5cm, LN > 2 cm, vascular involvement or impingement on major vessels (SMA, SMV, PV or HA), invasion of adjacent structures | RECIST | 1 - 5 | 50.4 Gy | NCI CTCAE v3 | 5/11 (45.4%) |  |
| 51 | Cho et al | Original | Pancreatology | 2013 | Retrospective | Yonsei University College of Medicine, Seoul, Korea | January 2002 - December 2011 | Gem + Cis/Cape | Yes | 13/30 (43.3%) | Mean 59.59, sd 8.57 | Male 16/30 (53.3%) | MD Anderson criteria | RECIST | 1 Cycles concurrent with radiation | 45 - 58.4 Gy | Not mentioned | 15/30 (50%) | Median follow up 26.7 months |
| 52 | Chuong et al BR | Original | International Journal of Radiation Oncology | 2013 | Retrospective | H Lee Moffitt Cancer Center, Tampa | June 2009 - December 2011 | Multiple | Yes | 6/32 (18.8%) | Median age 64 (38-87) | Male 36/73 (78.1%), Female 37/73 (21.9%) | NCCN guidelines | Other | Not mentioned | 35 Gy | NCI CTCAE v4 | 61/73 (83.6%) | Median 11 |
|  | Chuong et al LA |  |  |  |  |  |  |  |  |  |  |  |  |  |  |  |  |  | Median 7.8 |
| 53 | Faris et al | Original | The Oncologist | 2013 | Retrospective | Massachusetts General Hospital, Boston | July 2010 - February 2012 | FOLFIRINOX | Yes | - | Median 63 (range 45 - 78) | Male 13/22, female 9 /22 | NCCN guidelines | RECIST 1.1 | Median 8 (range 6 - 8) | Median 50.4 Gy | grading criteria not mentioned | Not mentioned | Median follow up 19.3 months |
| 54 | Gunturu et al | Original | Med Oncol | 2013 | Retrospective | Yale Cancer Center, New Haven | June 2010 - July 2011 | FOLFIRINOX | No | - | Median 61 (48 - 77) | Male 13/35 (37%), Female 22/37 (63%) | Not mentioned | RECIST | Median 11 | NA | Grading criteria not mentioned | Not mentioned |  |
| 55 | Gurka et al | Original | Radiation Oncology | 2013 | Prospective | Georgetown University, Washington DC | September 2009 - February 2011 | Gem | Yes | - | Median 62.5 range (50 -79) | Male 5/10 (50%), Female 5/10 (50%) | Other: > 180 degree encasement of the celiac, superior mesenteric, hepatic or gastroduodenal arteries, or greater than 2 cm involvement of the portal or SMV or any venous thrombus | RECIST | 6 | 25 Gy | NCI CTCAE v3 | NA |  |
| 56 | Kim et al | Original | Cancer | 2013 | Phase II study | Multi-institutional, US | July 2007 - February 2010 | GEMOX | Yes | 17/43 (40%) | Median 64 (range 42 - 83) | Male 32 (47%), Female 36 (53%) | NCCN guidelines | RECIST | Planned 4 cycles | 30 Gy | Grade 3 or higher reported. Criteria not mentioned | 26/68 (68%) |  |
| 57 | Leone et al | Original | Cancer | 2013 | Prospective series | University of Turin Institute of Cancer Research and Treatment, Candiolo, Italy | June 2002 - December 2009 | GEMOX | Yes | - | Median 63 (range 43 - 75) | Male 21/39 (53.8), Female 18/39 (46.2) | AHPBA/SSO/SSAT consensus criteria | RECIST | Median 4 | 50.4 Gy | NCI CTCAE v3 | Not mentioned | Median follow up of 12.9 months |
| 58 | Mahaseth et al | Original | Pancreas | 2013 | Retrospective | Emory University, Atlanta | June 2010 - June 2012 | FOLFIRINOX | Yes | - | Median 63 (range 36 - 78 ) | Male 26/60 (43%), Female 34/60 (57%) | Not mentioned | RECIST 1.1 | Median 3 cycles | Not mentioned | NCI CTCAE v4 | Not mentioned |  |
| 59 | Rajagopalan et al | Original | Radiation Oncology | 2013 | Retrospective | University of Pittsburgh Cancer Institute, Pittsburgh | 2008 - 2011 | Gem or Cape | Yes | 25% (4/12) | Median 68 (range 41 - 82) | Male 41.7% 5/12) | MD Anderson criteria | NA | Variable | 33 Gy | Not mentioned | 75% |  |
| 60 | Shinoto et al | Original | Cancer | 2013 | Phase I | Multi-institutional, Japan | April 2003 - December 2010 | None | Yes | - | Median 66 (range 40 - 79) | Male 15/26 (57.7%), Female 11/26 (42.3%) | Other criteria - no involvement of hepatic artery, celiac trunk or SMA, without evidence of metastatic disease. Involvement of portal vein, smv or splenic vein was not a contraindication | RECIST | NA | Mean 33.7 Gy | NCI CTCAE v2 | Not mentioned | Median 33.8 months |
| 61 | Sho et al | Original | J of Hepatobiliary Pancreat Sci | 2013 | Retrospective | Nara Medical University, Kashihara, Japan | September 2008 - October 2011 | Gem | Yes | 32/61 (52%) | Median 65.1 (range 36 - 78) | Male 36/61 (51%), female 25/61 (49%) | NCCN guidelines | RECIST | 1 Cycles concurrent with radiation | 50 - 54 Gy | NCI CTCAE v3 | 58/61 (95%) |  |
| 62 | Tajima et al | Original | Molecular and Clinical Oncology | 2013 | Phase I study | Kanazawa University, Ishikawa, Japan | October 2009 - May, 2012 | Gem + S1 | No | - | Median 64.5, range (38 - 74) | Male 6 Female 7 | Not mentioned | RECIST | 2 cycles | NA | NCI CTCAE v4 | Not mentioned |  |
| 63 | Tinchon et al | Original | Acta Oncol | 2013 | Retrospective | General Hospital Loeben, Loeben, Austria | 2010 - 2012 | FOLFIRINOX | No | 4/10 (40%) | Not mentioned | Not mentioned | AHPBA/SSO/SSAT consensus criteria | RECIST 1.1 | 4-6 | NA | NCI CTCAE v3 | Not mentioned | Median 15.4 months |
| 64 | Arvold et al 1 Chemo + CRT | Original | Cancer | 2012 | Retrospective | Massachusetts General Hospital, Boston | July 2005 - November 2009 | Multiple | Yes | - | Median 62 (range 44 - 87) | Male 16 (53.3%), female 14 (46.7%) | Other : Unresectable - tumors involving the celiac axis or SMA or occluding the SV or splenoportal confluence, Borderline: Tumors without complete vessel encasement but with substantial vascular encroachment. Definitions per surgical oncologist | Attending radiologist's report | Median 4 cycles | 50.4 Gy | NCI CTCAE v3 | 12/30 (40%) | Median 14.2 months |
|  | Arvold et al 2 CRT | Original | Cancer | 2012 | Retrospective |  |  |  | Yes | - | Median 63 (range 46 - 84) | Male 20 (50%), female 20 (50%) | Other : Unresectable - tumors involving the celiac axis or SMA or occluding the SV or splenoportal confluence, Borderline: Tumors without complete vessel encasement but with substantial vascular encroachment. Definitions per surgical oncologist | Attending radiologist's report | Concurrent with radiation | 50.4 Gy | NCI CTCAE v3 | 21/41 (51.2%) | Median 14.2 months |
| 65 | Barugola et al | Original | American Journal of Surgery | 2012 | Retrospective | University of Verona, Verona, Italy | January 2001 - December 2008 | Multiple | Yes | - | Median 59 (range 50 - 69) | Male 21/41 (51.2%), female 20/41 (48.8%) | MD Anderson criteria | NA | Concurrent with radiation | 45 - 50.4 Gy | NA | 32/41 (78%) |  |
| 66 | Bickenbach et al | Original | Annals of Surgical Oncology | 2012 | Retrospective | Memorial Sloan-Kettering Cancer Center, New York | January 2000 - December 2009 | Multiple | Yes | 7/36 (19%) | Median 61 (range 37 - 85) | Male 17/36 (47%) Females 19/36 (53%) | Other - Stage III pancreatic cancer |  |  |  |  |  |  |
| 67 | Habermehl et al | Original | Radiation Oncology | 2012 | Retrospective | Heidelberg, Germany | 2001 - 2010 | Multiple | Yes | - | Median 67 (range 42 - 93) | Male 110/215 (56%), female 88/215 (44%) | Similar to AHPBA/SSO/SSAT consensus | RECIST | Concurrent with radiation | Median 52.2 Gy | NCI CTCAE v4 | Not mentioned |  |
| 68 | Hosein et al | Original | BMC Cancer | 2012 | Retrospective | Sylvester Comprehensive Cancer Center, Miami | May 2008 - May 2011 | FOLFIRINOX | Yes | - | Median 57.5 (41 - 73) | Male 10/18 (56%), Female 8/18 (44%) | AHPBA/SSO/SSAT consensus criteria | RECIST | Median 8 cycles | Median 50.4 Gy | NCI CTCAE v4 | Not mentioned | Median follow up 13.4 months |
| 69 | Jesus-Acosta et al | Original | Cancer Chemother Pharmacol | 2012 | Retrospective | Multi-institutional, US | May 2003 - March 2010 | GTX | No | - | Median 62 (37 - 83) | Male 86 (56%), Female 68 (44%) | Not mentioned | RECIST | 4 | NA | NCI CTCAE v4 | Not mentioned | Not mentioned |
| 70 | Kang et al | Original | Journal of Gastrointestinal Surgery | 2012 | Retrospective | Yonsei University College of Medicine, Seoul, Korea | January 1999 - December 2010 | Gem | Yes | 13/32 (40.6%) | Mean 59.3, sd 9.1 | Male 14/32, female 18/32 | NCCN guidelines | WHO criteria | Concurrent with radiation | 45 or 50.4 Gy | NA | Not mentioned |  |
| 71 | Lee et al | Original | Surgery | 2012 | Prospective | University of Ulsan College of Medicine, Seoul, Korea | August 2006 - November 2008 | Gem or Cape | No | 14/17 (82.3%) | Median 61 (range 42 - 76) | Male 25/43 (58.1%), Female 18/43 (41.9%) | NCCN guidelines | RECIST 1.1 | Median 3 | 60 Gy | NCI CTCAE v3 | 12/14 (85.7%) | Median follow up 42.7 months |
| 72 | Papalezova et al | Original | Journal of Surgical Oncology | 2012 | Retrospective | Duke University Medical Center, Durham | 1999 - 2007 | 5FU | Yes | 14/76 (18%) | Mean 64, sd 12 | Male 78/144 (54%) | Other 1) Absence of extra pancreatic disease 2) no evidence of tumor extension to the SMA, celiac axis or hepatic artery as defined by a tissue plane between tumor and these arteries 3) patent SMV and PV | Not mentioned | Concurrent with radiation | 45 - 50.4 Gy | NA | 25/76 (33%) |  |
| 73 | Peddi et al | Original | Journal of Pancreas | 2012 | Retrospective | Multi-institutional, US | January 2009 - April 2012 | FOLFIRINOX | No | - | Median 58 (range 37 - 72) | Male 33/61 (54.1%), Female 28/61 (45.9%) | Not mentioned | RECIST | 4 (1 -22) | NA | NCI CTCAE v3 | Not mentioned | Median 8.5 |
| 74 | Tajima et al | Original | Experimental and Therapeutic Medicine | 2012 | Retrospective | Kanazawa University, Ishikawa, Japan | January 2006 - June 2009 | Gem + S1 | No | - | Median 62.6 (range 51 - 77) | Male 7/13, female 6/13 | Not mentioned | RECIST | 2 cycles | NA | grading criteria not mentioned | Not mentioned | Median 30 months |
| 75 | Takahashi et al | Original | Annals of Surgery | 2012 | Retrospective | Osaka University, Osaka, Japan | 2002 - 2009 | Gem | Yes | - | Age <= 65 63 (57%), Age >65 - 47 (43%) | Male 71/110 (65%), Female 39/110 (35%) | Other: UICC T3 7th edition | Not mentioned | Concurrent with radiation | 50Gy | NA | 4 weeks adjuvant therapy. % not mentioned |  |
| 76 | Loehrer et al Chemo | Original | Journal of Clinical Oncology | 2011 | Phase III | Multi-institutional, US | April 2003 - December 2005 | Gem | No | - | Median 69 (range 49.7 - 83.7) | Male 18/37(48.6%), Female 19/37 (51.4%) | Other - Surgical consultation - SMV or PV occlusion, SMA or HA encasement, gross regional lymphadenopathy | RECIST | 3 | NA |  |  | Not mentioned |
|  | Loehrer et al CRT |  |  |  |  |  |  | Gem | Yes | - | Median 66 (range 46.9 - 83.5) | Male 19/34 (55.9%), Female 15/34 (44.1%) | Other - Surgical consultation - SMV or PV occlusion, SMA or HA encasement, gross regional lymphadenopathy | RECIST | 3 | Mean 45.9 Gy | NCI CTCAE | Not mentioned | Not mentioned |
| 77 | Milandri et al | Original | Hepatogastroenterology | 2011 | Phase II study | Morgagni-Pierantoni Hospital, Forli, Italy | November 2004 - July 2008 | GEMOX | Yes | - | Median 64 (range 40 - 75) | Male 15/33 (45%), Female 18/33 (55%) | Other: invasion of peripancreatic organs other than duodenum, celiac axis or superior mesenteric artery involvement, occlusion of superior mesenteric or portal vein | RECIST | Median 6 | 25 Gy | WHO toxicity criteria | None | Median 19 months |
| 78 | Patel et al | Original | Journal of Surgical Oncology | 2011 | Retrospective | H. Lee Moffitt Cancer Center, Tampa | February 2006 - February 2009 | GTX | Yes | - | Median 67 (range 45 - 82) | Male 12/18 (66.7%) female 6/18 (33.3%) | NCCN guidelines | Not mentioned | Median 3 | Median 50 Gy | Not graded | Not mentioned | Median follow up of 13.27 months in patients who are still alive |
| 79 | Sahora NeoGemOx et al | Original | Surgery | 2011 | Phase II study | University of Vienna, Vienna, Austria | March 2003 - April 2006 | GEMOX | No | 5/13 (38.5%) | Median 61 (range 34 - 79) | Male 17/33 (51.5%) | Other: abutment <= 180 degree or encasement > 180 degree contact of the PV, SMV, celiac trunk and common hepatic artery, organ infiltration other than duodenum or stomach | RECIST | 6 | NA | NCI CTCAE | None |  |
| 80 | Sahora NeoGemTax et al | Original | World J Surg | 2011 | Phase II | University of Vienna, Vienna, Austria | June 2001 - December 2003 | Gem + Docetaxel | No | 2/8 (25%) | Median 61 (range 34 - 79) | Male 11/25 (44%), Female 14/25 (56%) | AHPBA/SSO/SSAT criteria | RECIST | 2 | NA | NCI CTCAE | Not mentioned |  |
| 81 | Schellenberg et al | Original | International Journal of Radiation Oncology | 2011 | Retrospective | Stanford University, Stanford | April 2006 - October 2007 | Gem | Yes | - | Median 63 (range 45 - 85) | Male 9/20 (45%), Female 11/20 (55%) | Other: > 180 degree involvement of SMA/SMV or any involvement of celiac axis, portal vein occlusion, aorta or IVC invasion | RECIST | 5 | 25 Gy | NCI CTCAE v3 | Not mentioned | Up to 36 months |
| 82 | Small et al | Original | International Journal of Radiation Oncology | 2011 | Phase II | Robert H Lurie Comprehensive Cancer Center of Northwestern University, Chicago | October 2005 - October 2007 | Gem + Bevacizumab | Yes | - | Median 62 (range 43 - 80) | Male 18/29 (62%), Female 11/29 (38%) | NCCN guidelines | RECIST 1.0 | 3 | 36 Gy | NCI CTCAE v3 | Not mentioned | Median 11.1 months |
| 83 | Stokes et al | Original | Annals of Surgical Oncology | 2011 | Retrospective | University of Virginia, Charlottesville | August 2005 - August 2008 | Cape | Yes | 4/16 (25%) | Median 66 (range 45 - 83) | Male 25/40 (63%), Female 15/40 (37%) | MD Anderson criteria | Not mentioned | Concurrent with radiation | 50.4 Gy | RTOG radiation morbidity score | 14/16 (88%) | Median follow up of 13 months in 10 alive patients |
| 84 | Katz et al | Original | Annals of Surgical Oncology | 2010 | Retrospective analysis of phase II studies | MD Anderson Cancer Center, Houston | 1998 - 2006 | Gem + Cis | Yes | - | Median 64 (range 38 - 80) | Male 104/174 (60%), Female 70/174 (40%) | Other : No evidence of extra pancreatic disease, absence of tumor extension to the SMA or celiac axis, No evidence of occlusion of SMV or SMV-PV confluence | Not mentioned | variable | variable | NA | Not mentioned | Median follow up of surviving patients 78 months |
| 85 | Landry et al 1 Arm A | Original | Journal of Surgical Oncology | 2010 | Phase II Double armed | Multi-institutional, US | October 2003 - June 2005 |  | Yes | - | Median 53.6 | Male 6/10 (60%), Female 4/10 (40%) | Other: Potentially resectable - abutting the portal or SMV, abutting the hepatic or superior mesenteric artery, extending to the origin of gastroduodenal artery, occluding SMV < 2cm, locally advanced, or explored and deemed unresectable | RECIST | 1 cycle concurrent with radiation (6 weekly doses) | 50.4 Gy | grading criteria not mentioned | 2/3 (66.7%) |  |
|  | Landry et al 2 Arm B | Original | Journal of Surgical Oncology | 2010 | Phase II Double armed |  |  | Gem + Cis | Yes | - | Median 53.6 | Male 6/11 (54.5), Female 5/11 (45.5) | Other: Potentially resectable - abutting the portal or SMV, abutting the hepatic or superior mesenteric artery, extending to the origin of gastroduodenal artery, occluding SMV < 2cm, locally advanced, or explored and deemed unresectable | RECIST | 1 cycle | 50.4 Gy | grading criteria not mentioned | 1/2 (50%) |  |
| 86 | Mahadevan et al | Original | International Journal of Radiation Oncology | 2010 | Retrospective | Beth Israel Deaconess Medical Center, Boston | September 2005 - October 2007 | Gem | Yes | - | Median 65 (range 43 - 88) | Male 26/36 (72.2%), Female 10/36 (27.8%) | NCCN guidelines | None | 6 | 30 Gy | grading criteria not mentioned | NA | Median 24 |
| 87 | Mattiucci et al | Original | International Journal of Radiation Oncology | 2010 | Phase II | Catholic University, Rome, Italy | January 2000 - June 2005 | Gem | Yes | - | Mean 62 (range 36 - 76) | Male 22/40 (55%) Female 18/45 (45%) | Tumors with invasion of major regional vessels or neighboring organs (except for duodenum and primary bile duct) | WHO criteria | Concurrent with radiation | 50.4 Gy | RTOG criteria | Not mentioned | Median 76 |
| 88 | McClaine et al | Original | HPB | 2010 | Retrospective | University of Cincinnati, Cincinnati | 2003 - 2008 | Multiple | Yes | 5/12 (42%) | Mean 63 | Male 14/29 (48.3%), Female 15/29 (51.7%) | NCCN and MD Anderson criteria | Not mentioned | Variable | Not mentioned | Not mentioned | 4/9 (44.4%) |  |
| 89 | Polistina et al | Original | Annals of Surgical Oncology | 2010 | Prospective non randomized | San Bortolo Hospital, Vicenza, Italy | August 2004 - May 2007 | Gem | Yes | 1/3 (33%) | Median 68 (range 44 - 75) | Male 15/23 (65.2%), Female 8/23 (34.8%) | Other: Mass involving the celiac axis, with > 2 cm circumferential invasion of SMA, or both, involvement of intramesocolic portion of SMV down to the confluence of jejunal veins | RECIST | 6 week course of Gemcitabine | 30 Gy | NCI CTCAE v3 | Not mentioned | Median follow up 9 months |
| 90 | Turrini et al | Original | European Journal of Surgical Oncology | 2010 | Phase II study | Insitut Paoli-Calmettes and Univeriste de la Mediterranee, Marseille, France | May 2003 - July 2005 | Gem + Docetaxel | Yes | 5/17 (29.4%) | Mean 61.5 (range 40 -72) | Male 21/34 (61.8%), Female 13/34 (38.2%) | Other: Excluded patients include tumors involving > 180 degree of the PV or SMV, occlusion of SMV or PV confluence or direct tumor extension to either the SMA or Celiac axis or any evidence of extrapancreatic disease | RECIST | Concurrent with radiation | 45 Gy | WHO toxicity criteria | Not mentioned | Median follow up 54 months |
| 91 | Bjerregaard et al | Original | Radiotherapy and Oncology | 2009 | Retrospective | Odense University Hospital, Odense, Denmark | 2001 - 2005 | UFT | Yes | - |  |  | - |  |  |  |  |  |  |
| 92 | Le Scodan et al | Original | Annals of Oncology | 2009 | Phase II | Hospital Edouard Herriot, Lyon, France | January 1998 - March 2003 | 5FU and Cisplatin | Yes | - | Median 59.3 (range 33 - 75) | Male 25/41 (61%), Female 16/41 (39%) | Other - AJCC stage 1, 2 and 3 pancreatic cancer | WHO criteria | Concurrent with radiation | 50 Gy | WHO criteria | Not mentioned | Median 11 months |
| 93 | Maixmous et al | Original | International Archives of Medicine | 2009 | Retrospective | Assiut University, Assuit, Egypt | March 2006 - November 2007 | Gem | Yes | - | Median 46 (range 40 - 68) | Male 15/25 (60%) Female 10/25 (40%) | T4 tumors - celiac or superior mesenteric artery encasement | WHO criteria | Concurrent with radiation | 54 Gy | WHO toxicity criteria | Not mentioned | Not mentioned |
| 94 | Satoi et al 1 R | Original | Pancreas | 2009 | Retrospective | Kansai Medical University, Osaka, Japan | January 2000 - December 2005 | Multiple | Yes | - | Median 64 (range 47-74) | Male 37%, Female 63% | NCCN guidelines | Not mentioned | Concurrent with radiation | 40 Gy | NA | Not mentioned | Median follow up 20.5 months |
|  | Satoi et al 2 BR | Original | Pancreas | 2009 | Retrospective |  |  |  |  | 22% | Median 64 (range 47-74) | Male 37%, Female 63% | NCCN guidelines | Not mentioned | Concurrent with radiation | 40 Gy | NA | Not mentioned |  |
| 95 | Tinkl et al | Original | Strahlentherapie und Onkologie | 2009 | Retrospective | University of Erlangen-Nurember, Erlangen, Germany | September 1996 - February 2006 | Multiple | Yes | - | Median 61 (range 41 - 76) | Male 24/38 (63%), Female 14/38 (37%) | NCCN guidelines | NA | Concurrent with radiation | 106.2 Gy | NCI CTCAE v3, RTOG | Not mentioned |  |
| 96 | Wilkowski et al 1 Arm A | Original | British Journal of Cancer | 2009 | Phase II | Multicenter, Germany | February 2002 - July 2005 | Gem + Cis | Yes | - | Median 63 (42 - 74) | Male 15/31 (48%), Female 16/31 (52%) | Stage III and IVA | WHO criteria | Concurrent with radiation | 50 Gy | WHO criteria | Not mentioned | Median 8.6 |
|  | Wilkowski et al 2 Arm B | Original | British Journal of Cancer | 2009 | Phase II |  |  |  | Yes | - | Median 63 (40 - 75) | Male 16/32 (50%), Female 16/32 (50%) | Stage III and IVA | WHO criteria | Concurrent with radiation | 50Gy | WHO criteria | Not mentioned | Median 8.6 |
|  | Wilkowski et al 3 Arm C | Original | British Journal of Cancer | 2009 | Phase II |  |  |  | Yes | - | Median 65 (41 - 75) | Male 20/31 (65%), Female 11/31 (35%) | Stage III and IVA | WHO criteria | Median 2 | 50 Gy | WHO criteria | Not mentioned | Median 8.6 |
