# Supplementary material for: Neoadjuvant treatment of pancreatic adenocarcinoma: a systematic review and meta-analysis of 5520 patients
Source: World J Surg Oncol. 2017 Oct 10;15:183. doi: 10.1186/s12957-017-1240-2 (PMC5634869; doi:10.1186/s12957-017-1240-2)
Supplement: Supplementary file 2 — Pathologic response to neoadjuvant therapy (DOCX 17 kb) [file 12957_2017_1240_MOESM2_ESM.docx]

| Additional file 2: Table 2. Pathologic response to neoadjuvant therapy | | | |
| --- | --- | --- | --- |
| Evans grade | n | % | 95% Confidence Interval |
| Grade 1 | 13 | 12% | 6% - 20% |
| Grade 2a | 13 | 37% | 24% - 51% |
| Grade 2b | 15 | 27% | 19% - 36% |
| Grade 3 | 18 | 13% | 6% - 22% |
| Grade 4* | 30 | 3% | 1% - 5% |
| *** includes all pathologic complete responses. n – number of studies reporting the outcome**. | | | |
