# Supplementary material for: Neoadjuvant treatment of pancreatic adenocarcinoma: a systematic review and meta-analysis of 5520 patients
Source: World J Surg Oncol. 2017 Oct 10;15:183. doi: 10.1186/s12957-017-1240-2 (PMC5634869; doi:10.1186/s12957-017-1240-2)
Supplement: Supplementary file 3 — Funnel plots for assessment of publication bias and small study (PPTX 88 kb) [file 12957_2017_1240_MOESM3_ESM.pptx]

## Slide 1
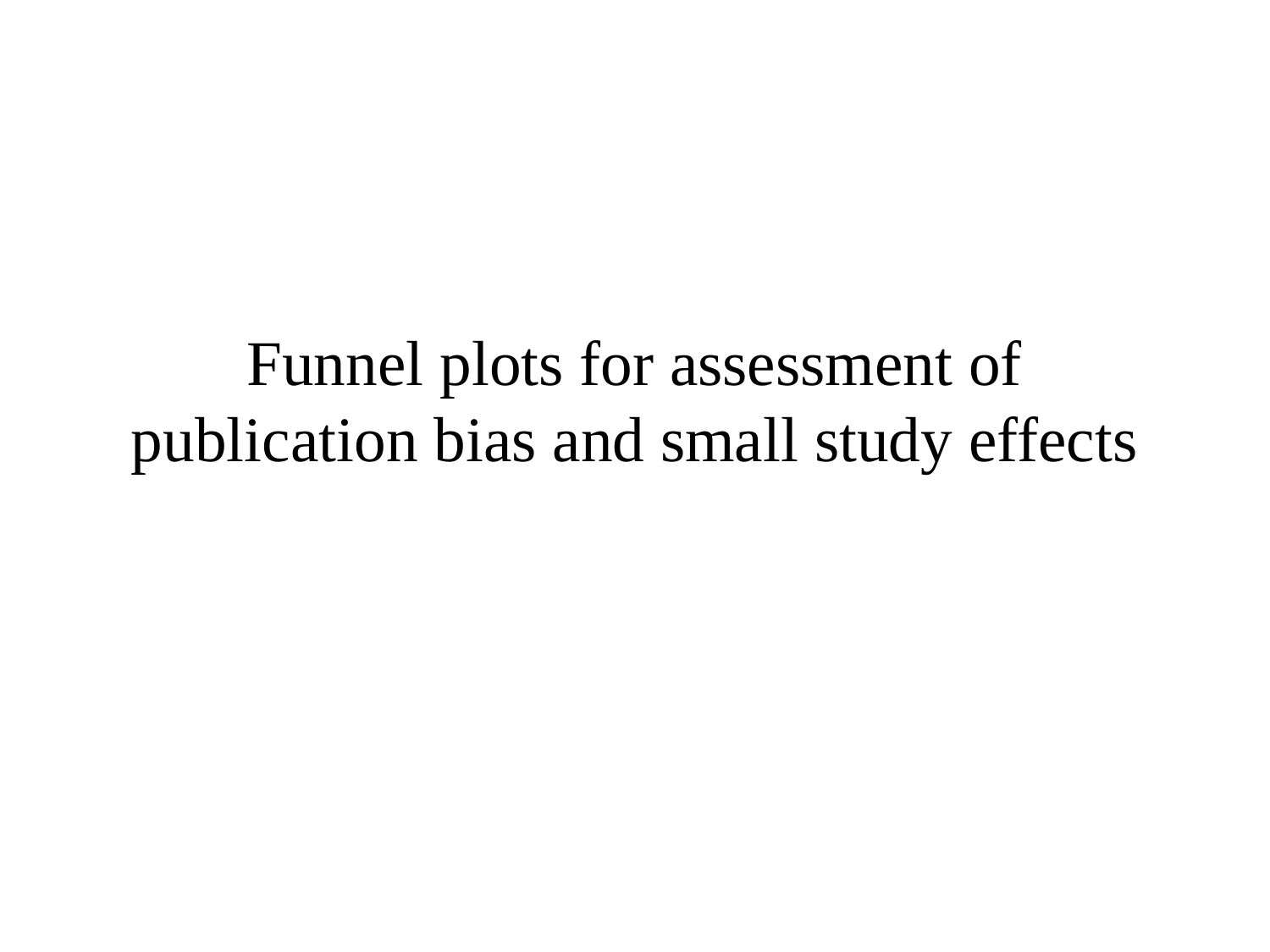

# Funnel plots for assessment of publication bias and small study effects

## Slide 2
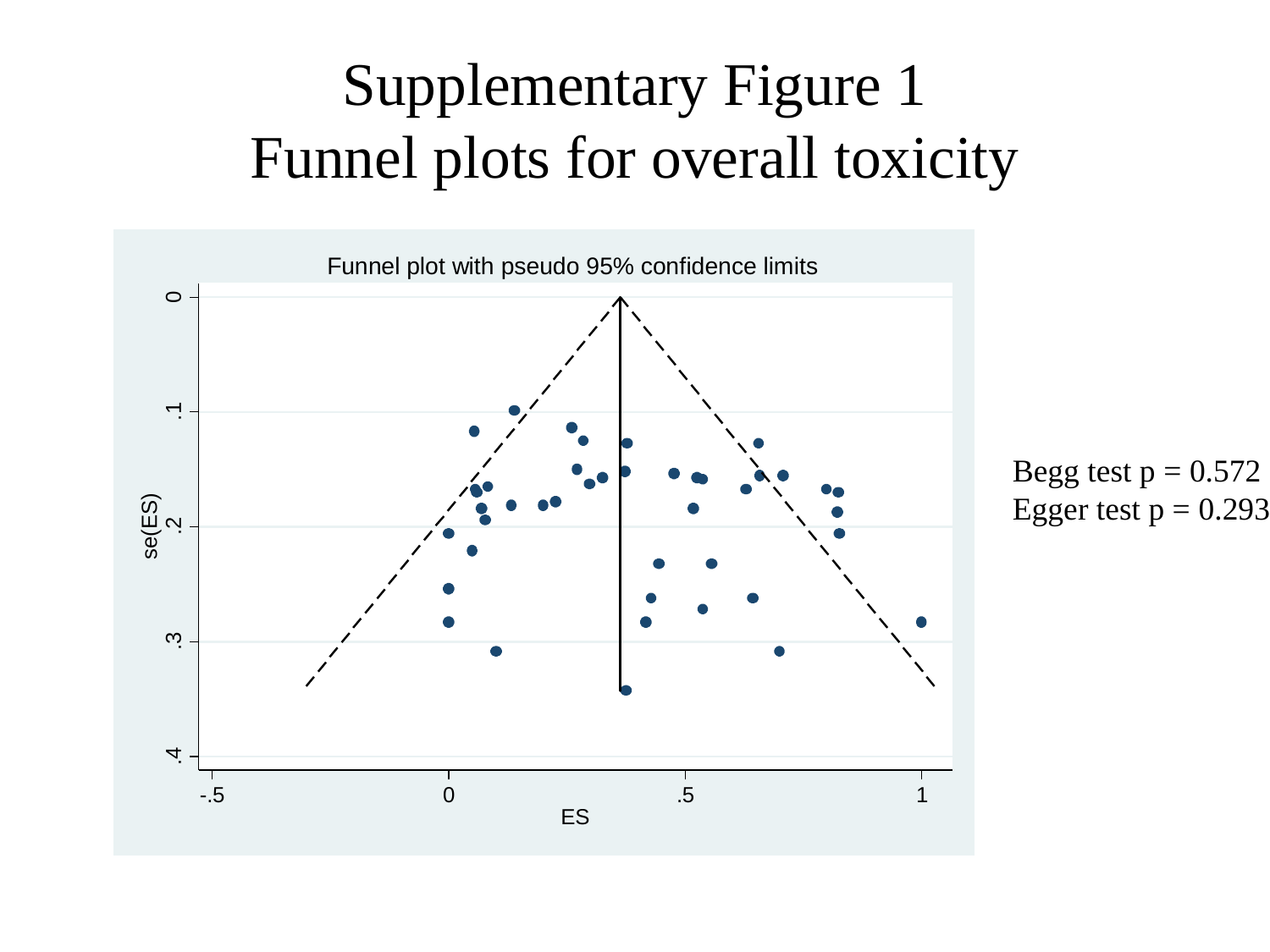

# Supplementary Figure 1 Funnel plots for overall toxicity
Begg test p = 0.572
Egger test p = 0.293

## Slide 3
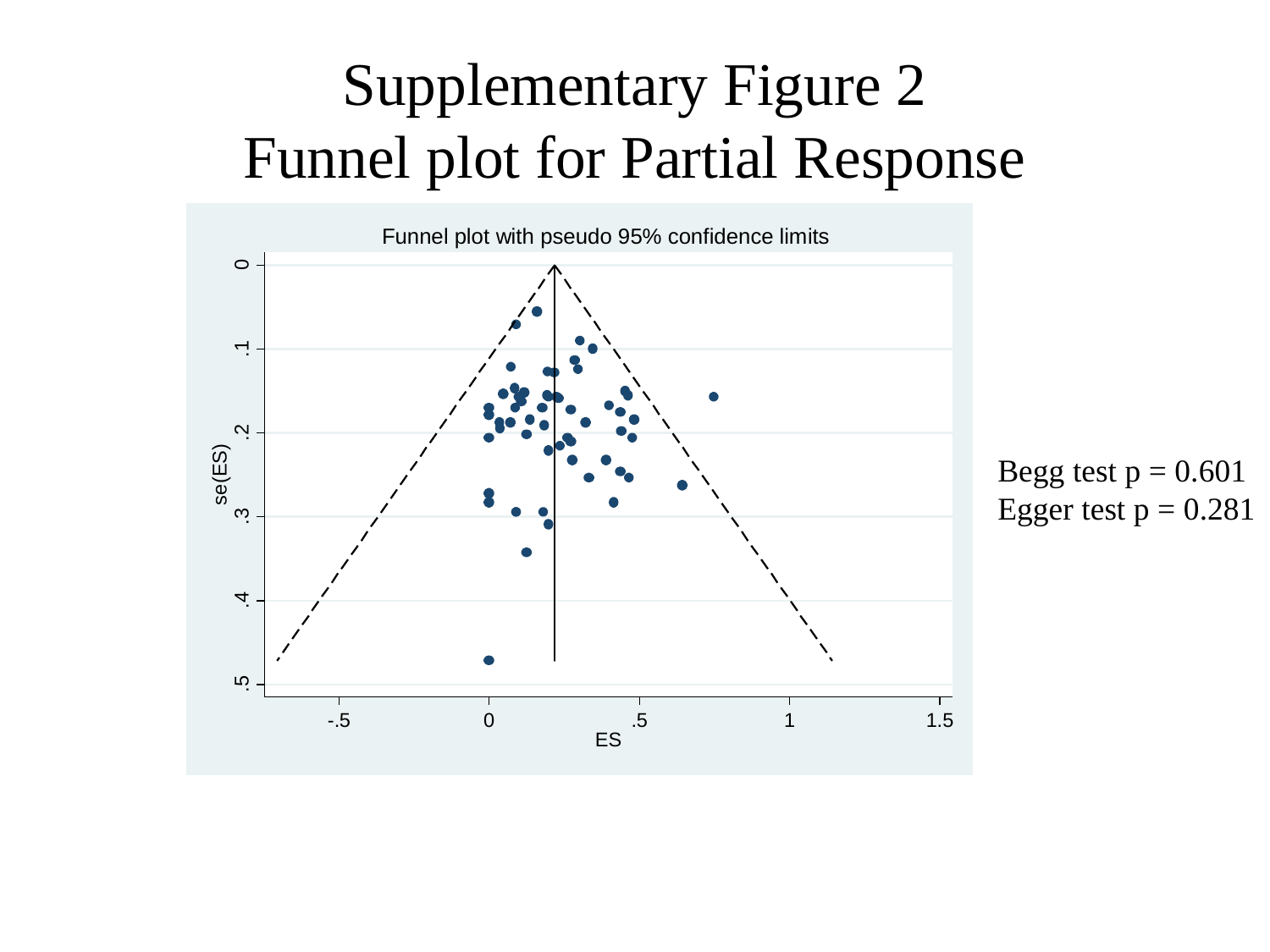

# Supplementary Figure 2 Funnel plot for Partial Response
Begg test p = 0.601
Egger test p = 0.281

## Slide 4
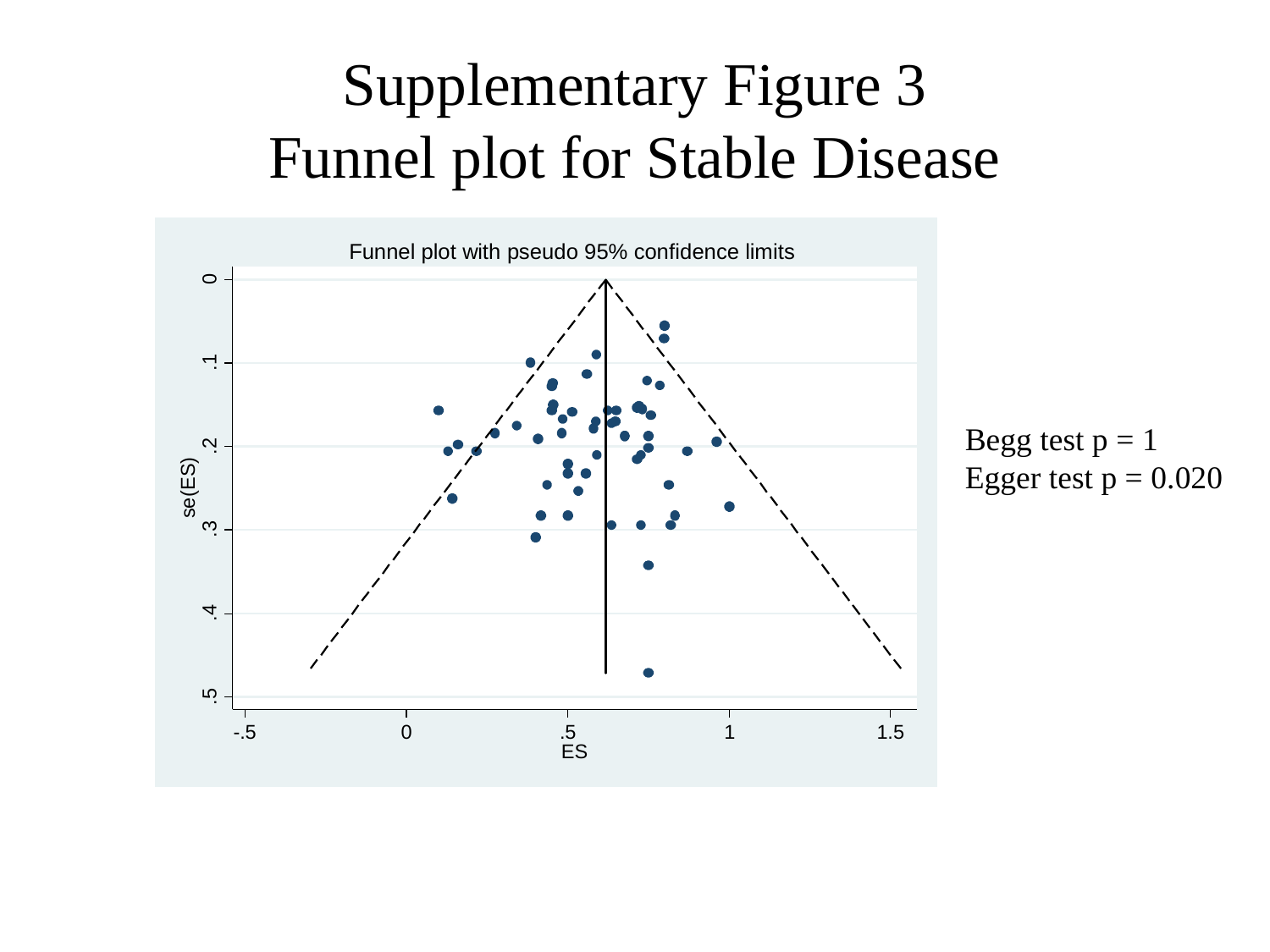

# Supplementary Figure 3 Funnel plot for Stable Disease
Begg test p = 1
Egger test p = 0.020

## Slide 5
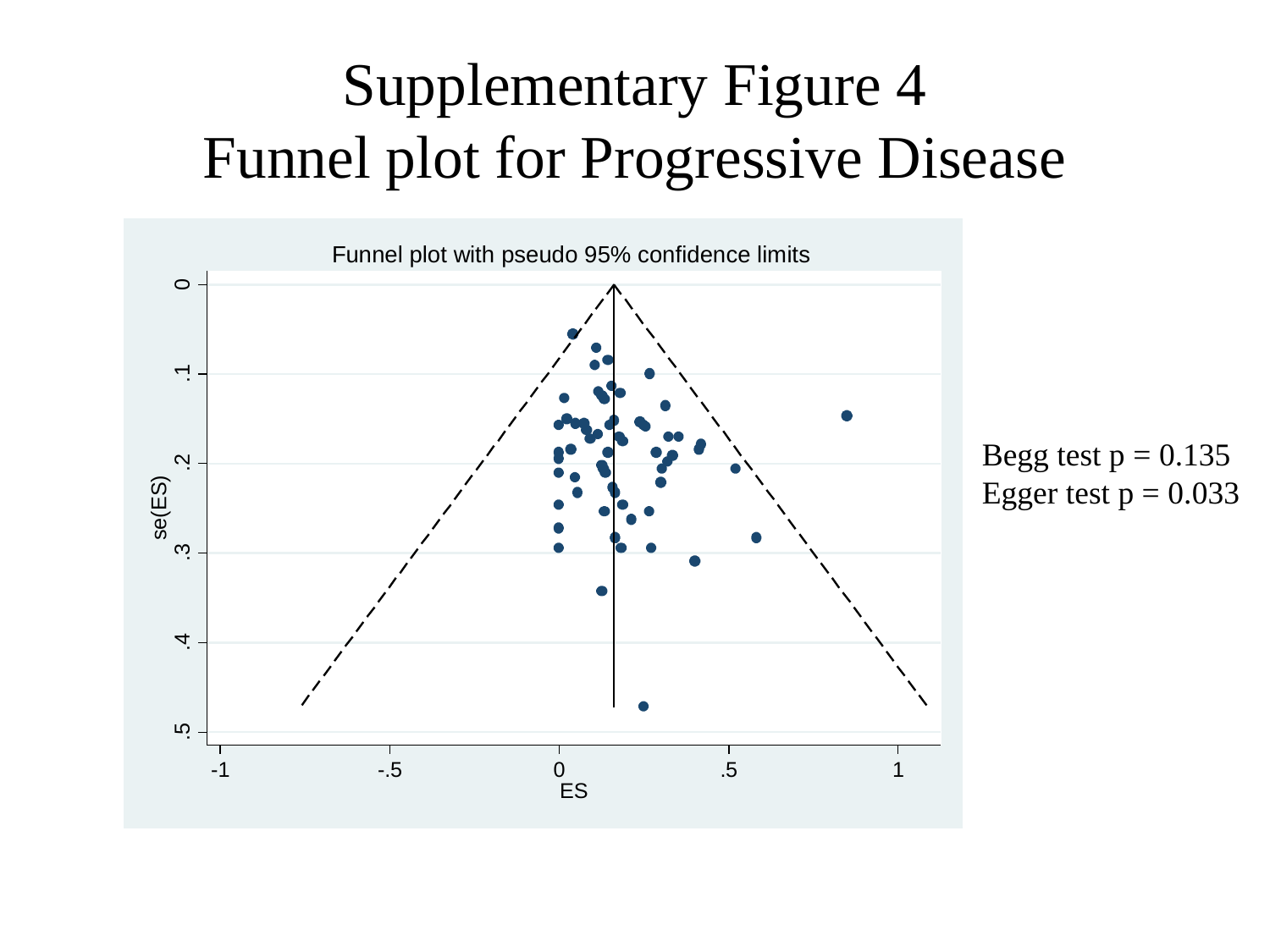

# Supplementary Figure 4 Funnel plot for Progressive Disease
Begg test p = 0.135
Egger test p = 0.033

## Slide 6
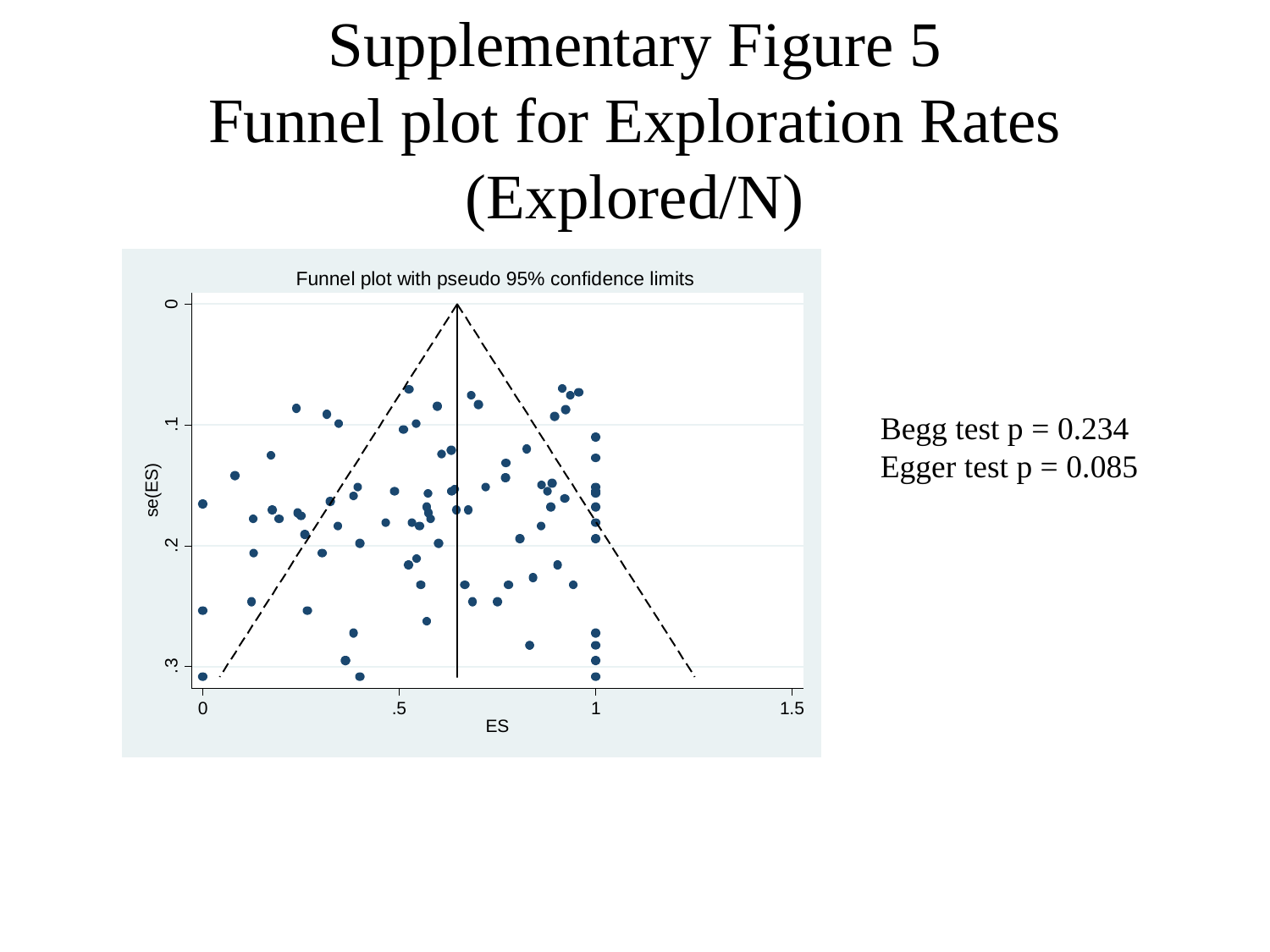

# Supplementary Figure 5 Funnel plot for Exploration Rates (Explored/N)
Begg test p = 0.234
Egger test p = 0.085

## Slide 7
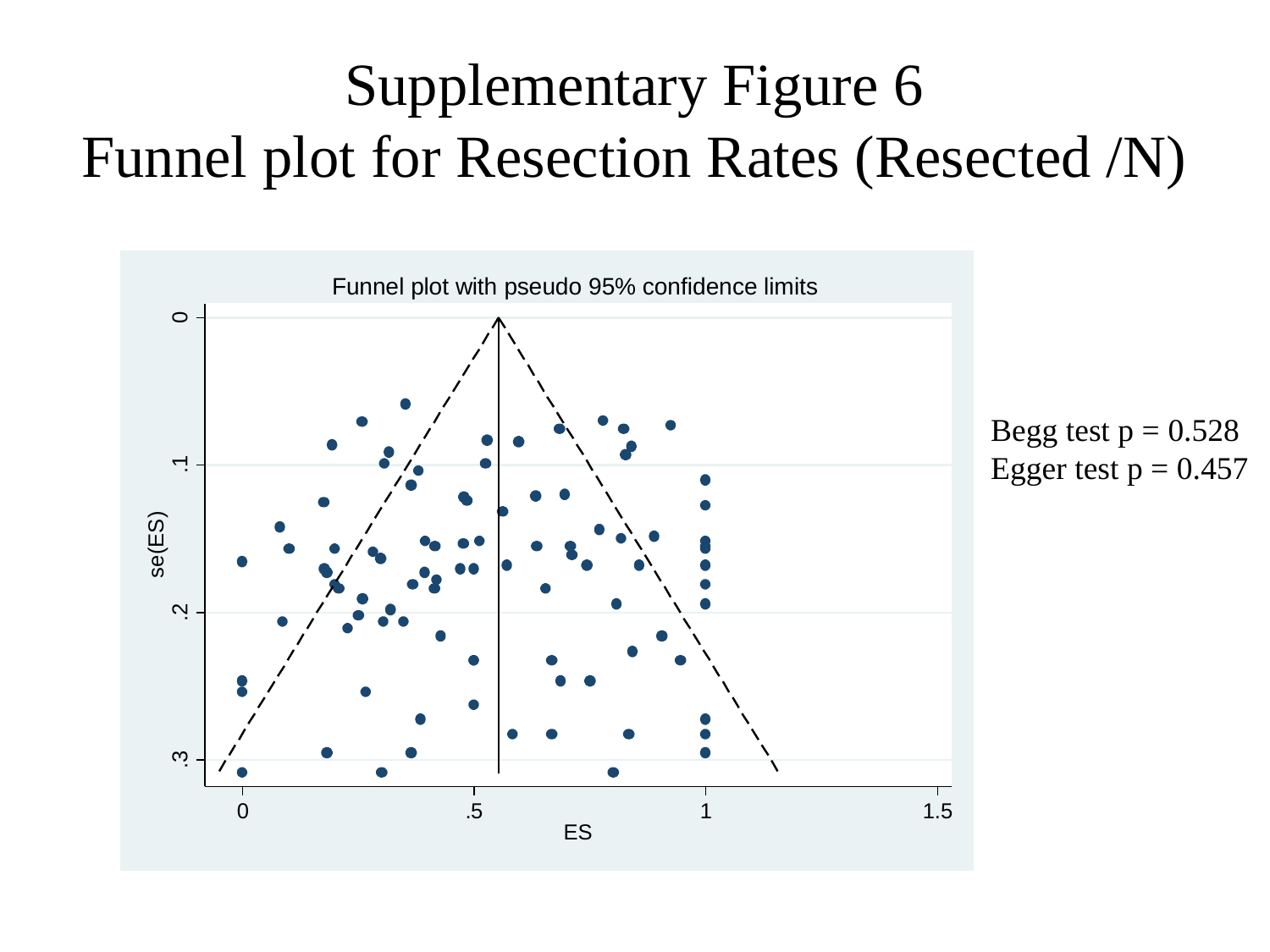

# Supplementary Figure 6 Funnel plot for Resection Rates (Resected /N)
Begg test p = 0.528
Egger test p = 0.457

## Slide 8
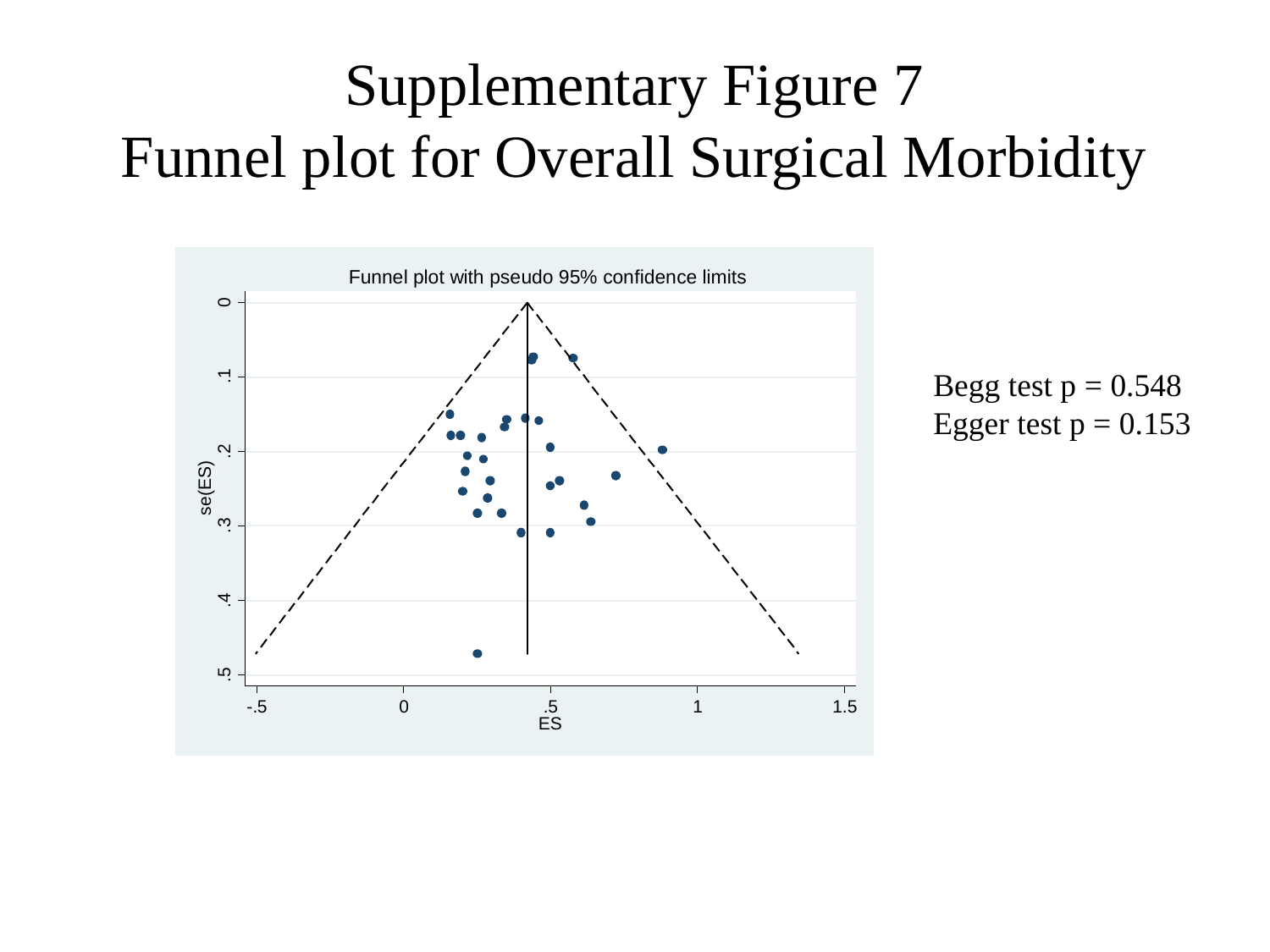

# Supplementary Figure 7 Funnel plot for Overall Surgical Morbidity
Begg test p = 0.548
Egger test p = 0.153

## Slide 9
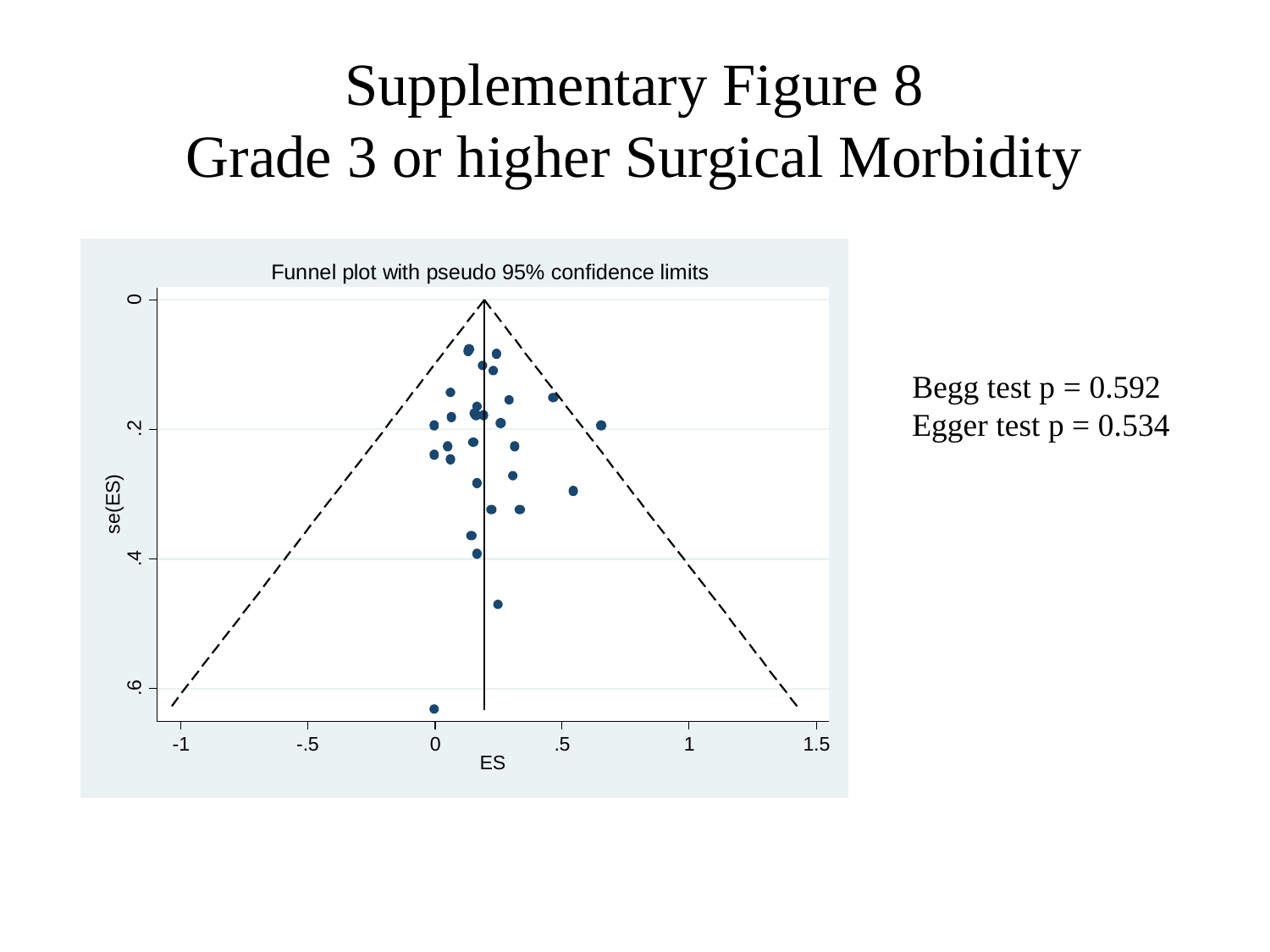

# Supplementary Figure 8 Grade 3 or higher Surgical Morbidity
Begg test p = 0.592
Egger test p = 0.534
